# Gestational diabetes and stillbirth: a systematic review and meta-analysis

**DOI:** 10.1016/j.eclinm.2025.103751

**Published:** 2026-01-08

**Authors:** Victoria Bradley, Livia Samara, Miriam Toolan, Amelia Atkinson, Deborah M. Caldwell, Abigail Fraser, Shakila Thangaratinam, Gemma Clayton, Abi Merriel

**Affiliations:** a Centre for Women's Health Research, Department of Women's and Children's Health, Institute of Life Course and Medical Sciences, University of Liverpool, United Kingdom; b NIHR North West Coast Applied Research Collaboration, United Kingdom; c University Hospitals Bristol and Weston, United Kingdom; d Population Health Sciences, Bristol Medical School, Bristol, United Kingdom; e CDHB Health, Te Whatu Ora, New Zealand; f MRC Integrative Epidemiology Unit at the University of Bristol, Bristol, United Kingdom; g Liverpool Women's Hospital NHS Foundation Trust, Liverpool, United Kingdom

**Keywords:** Gestational diabetes, Stillbirth, Obstetrics

## Abstract

**Background:**

Globally, around 2 million pregnancies each year end in stillbirth, with the majority occurring in low and middle-income countries. The association between gestational diabetes mellitus (GDM) and stillbirth has been widely researched but the evidence is increasingly controversial. The aim of this study is to evaluate the risk of stillbirth associated with GDM, and examine whether this risk differs according to country income status and GDM screening method.

**Methods:**

We searched Scopus, Cinahl, Cochrane Central, ISRCTN, Medline, Embase and Epistemonikos on the 9th May 2025 from inception to May 2025 with no restrictions based on language, study location or time. We included cohort studies that estimated the association of interest. We conducted a random effects meta-analysis by study design and sub-group analyses by country income level and GDM screening method. (PROSPERO CRD4201800057).

**Findings:**

101 included studies (92,915,856 women) presented unadjusted results, 19 reported adjusted results (63,629,536 women). Meta-analysis of adjusted cohort data did not show evidence of an association between a diagnosis of GDM and the risk of stillbirth worldwide (OR 0.81, 95% CI: 0.68–0.97; I^2^ = 87.7%; n = 19 studies). However, when stratified by country income level a diagnosis of GDM was associated with a reduction in the odds of stillbirth in high income countries (OR 0.73, 95% CI: 0.65–0.82; I^2^ = 31.9%; n = 13 studies), but this was not observed in upper and lower middle income countries, (OR 1.17, 95% CI: 0.71–1.93; I^2^ = 59.7%; n = 6 studies). There were no adjusted estimates from low-income countries. There was no evidence of a difference by GDM screening method.

**Interpretation:**

Increased screening, timely diagnosis and effective management strategies for GDM in high-income countries, such as induction of labour and increased antenatal care, may be responsible for the reduced risk of stillbirth in women with GDM. Further research is needed to identify the optimum strategy for screening and management in low and middle-income settings to reduce preventable stillbirths worldwide.

**Funding:**

None.

**Research in context**

**Evidence before this study**

Gestational diabetes has long been believed to increase the risk of stillbirth, but recent literature suggests a more inconclusive relationship. Stillbirth occurs more frequently in low- and middle-income countries, where increased antenatal care and screening is not as robust or available. Women in high income countries are more often screened for gestational diabetes and those diagnosed receive increased antenatal care, scans and an earlier birth.

**Added value of this study**

This study aimed to examine gestational diabetes and stillbirth in the context of country income level. This study spanned a greater depth of literature than previous studies, with an increased number of low-and middle-income countries represented. This review found a diagnosis of gestational diabetes to be a protective factor against stillbirth in high-income countries and was inconclusive in low-and middle-income countries. There was no difference in outcome with universal vs risk-based screening worldwide.

**Implications of all the available evidence**

Increased antenatal care, earlier intervention and earlier delivery in pregnancies complicated with gestational diabetes may be contributing to a decreased risk of stillbirth in high income countries. Low- and middle-income countries may face a greater risk of stillbirth due to fewer resources to care for women with gestational diabetes. The current literature suggests the relationship between stillbirth and gestational diabetes is not clear cut, and may be confounded by the care and interventions women with gestational diabetes receive.

## Introduction

There are nearly 2 million stillbirths every year,^1^ which are life-altering for women and their families, with a significant long-term psychosocial impact.^2^ The majority of stillbriths occur in low-and-middle income (LMIC) countries,^3^ nearly half of which happen in Sub-Saharan Africa.^1^^,^^4^ In 2023, Western Europe and North America had fewer than 3 stillbirths per 1000 total births, while Sub-Saharan, West and Central Africa had estimated rates of more than 20 per 1000 births, with South Asia following closely behind at 16.3 per 1000 births.^4^^,^^5^ Under 3 stillbirths per 1000 births occur in high income countries, compared to 23.3 in low income countries.^4^ This economic variation becomes increasingly apparent within middle income countries where the stillbirth rate contrasts significantly between upper middle income countries (6.9 per 1000 births) and lower middle income countries (16.3 per 1000 births).^4^ This dramatic regional and economic variation demonstrates that the majority of stillbirths, are likely to be preventable.^6^

There are myriad reasons for stillbirth including maternal and foetal complications. One is gestational diabetes mellitus (GDM), a maternal disease, associated with poor foetal outcomes including macrosomia, neonatal admission and stillbirth.^7^, ^8^, ^9^ There is a clear association between undiagnosed, or poorly treated GDM and stillbirth.^9^ However, there is increasing questioning over the link between well managed GDM and stillbirth.^10^ This is becoming increasingly important, as GDM is estimated to affect one in six pregnancies worldwide.^11^ The prevalence has increased in recent years due to altered thresholds for GDM diagnosis,^12^^,^^13^ rising prevalence of obesity,^14^ reduced physical activity levels, and increasing maternal age.^15^

A diagnosis of GDM in high income settings can have a significant impact on a woman's pregnancy course resulting in increased antenatal contacts and timely delivery being recommended. Whilst in LMICs it is frequently under-diagnosed. Studies conducted to assess the relationship between GDM and stillbirth have yielded inconsistent results.^16^, ^17^, ^18^ A recent systematic review, with no restriction on country, concluded that there is not enough data to define a relationship between GDM and stillbirth (unadjusted OR 1.04 [95% CI: 0.90, 1.21]; I^2^ = 86%), although their estimate using adjusted OR suggested there may be an effect (0.78 [95% CI: 0.68, 0.88]; I^2^ = 43%). However, despite a signficant variation in diagnosis and management of GDM globally, they did not consider the impact of country income level or type of GDM screening on these estimates.^19^

The definition of GDM seems simple: *any degree of glucose intolerance with onset, or first recognition, in pregnancy*,^20^ however, screening methods to identify, and diagnostic criteria to confirm GDM vary greatly globally.^21^ Universal screening offers testing to all women; risk-based, or selective screening, involves only offering testing to those deemed at high-risk of GDM.^22^ However, the most appropriate method for screening for GDM is not clear.^22^

Our aim was to estimate the association between GDM and stillbirth, separately in high, middle and low-income settings and understand if the approach to screening for GDM impacts this association.

## Methods

We performed a systematic review and meta-analysis of studies investigating the association between GDM and stillbirth. The review protocol was registered on Prospero (ID CRD42018100057) where the protocol and amendments can be viewed. We have reported our findings following the Preferred Reporting Items for Systematic Reviews and Meta-Analyses (PRISMA) reporting guidelines.^23^

### Search methods

We searched Scopus, Cinahl, Cochrane Central, ISRCTN, Medline, Embase and Epistemonikos from inception to 5th May 2025. Searches were not restricted by language, location of study or time. We also reviewed the reference lists of all included studies for further eligible studies. Titles, abstracts and full texts were searched for terms including stillbirth, foetal death and gestational diabetes. The full search strategy can be found in the Supplementary Files.

### Study selection

Only cohort studies were included in this report due to their higher quality, compared to case–control studies, despite their observational nature. Studies were included if they reported rates of GDM and stillbirth. Studies only reporting data for women with pre-existing diabetes were excluded. We included any method of diagnosing GDM and any definition authors used for stillbirth, as long as foetal death was spontaneous, and occurred after 20 weeks gestation but prior to birth. We included studies from any setting and in any language. Conference abstracts were eligible for inclusion if they reported relevant estimates.

### Data extraction and risk of bias assessment

Study quality and risk of bias were evaluated using the Newcastle–Ottawa Quality Assessment Scale (NOS) for non-randomised studies. Two reviewers (LS/VB) independently assessed all criteria for NOS assessment. Assessment included consideration of strengths and weaknesses in four domains: selection of the participants, comparability of the groups, ascertainment of reported outcomes and quality of follow-up. Studies that adjusted for at least 2 of: maternal age, BMI and smoking were assessed as high quality (2 stars) for the group comparability domain and studies that controlled for any confounding factors were assessed as medium quality (1 star). Full details of the scale and assessment are available in Supplementary Table S1.

Abstract screening and full text review were performed using Covidence^24^ and in duplicate (AA/MT/LS/VB). Disagreements were resolved through discussion with a senior author (GC/AM). A standardised, pre-piloted data extraction form was used. We extracted the country, funding source, methodology, study duration alongside the definition of stillbirth used, and the screening method and criteria used to diagnose GDM. Country income level was assessed using the World Bank classification system, 2024.^25^ We extracted both unadjusted and adjusted effect estimates where available as odds ratios (ORs) or relative risks (RRs), along with the number of events and total sample size per group. Any adjusted odds ratio was accepted, regardless of the cofounders adjusted for. In studies where relevant data were not published, the authors were contacted.

### Statistical analysis

Meta-analyses were conducted for this review. We anticipated heterogeneity between studies (e.g. by setting, timing of GDM diagnosis, differences in definition of stillbirth) and therefore used random effects meta-analysis in R 4.4.1 (Meta),^26^ estimating between study heterogeneity (tau squared) using restricted maximum likelihood. The inverse variance weighted method was applied with the Hartung-Knapp adjustment. We described the percentage of variability in study estimates due to heterogeneity (I^2^) rather than chance.^27^^,^^28^ We used meta-regression to formally assess whether between-study heterogeneity in the association between GDM diagnosis and stillbirth was explained by study-level characteristics: country income level (high-income vs. upper/lower-middle-income) and screening approach (universal vs. risk-based vs not stated), separately.

To perform the meta-analysis, effect estimates and their standard errors (or equivalent) were needed from each study. Given that there were adjusted and unadjusted estimates reported, we report these separately favouring the adjusted results as they account for potential confounders. In unadjusted data, a continuity correction of 0.5 was applied to studies with zero events, to avoid issues with undefined values in studies where no events occurred in one of the groups. This continuity correction can introduce bias when event rates are low or study sizes are small. Therefore to explore this, we undertook a sensitivity analyses excluding those studies.^29^^,^^30^ The comparable results showed that the findings were not driven by methodological choices. We combined studies reporting either OR and RR, as these are approximately equivalent for rare events.^31^

Overall pooled summaries are reported in the main text as OR and 95% confidence intervals for adjusted data, with unadjusted results also presented in Supplementary Figures S1 and S2. Sub-group analyses were performed, as planned, by country income level (high income, (upper and lower) middle income, low income) and by type of screening (universal, risk-based, not stated).

Sensitivity analyses restricting to ‘good quality’ studies were conducted. We also used funnel plots to assess small-study effects, which refer to the tendency for smaller studies to show different, often exaggerated, effects compared to larger studies. We used Egger's test to assess funnel plot asymmetry, Egger's test is a linear regression method in which the effect sizes (log-transformed odds ratios divided by their standard errors) are regressed against their standard errors. We interpret the intercept of this regression as a measure of asymmetry: evidence of a non-zero intercept suggests that smaller studies report systematically different effects compared to larger studies.

### Role of the funding source

No funding was received for this study.

## Results

**Fig. 1:**
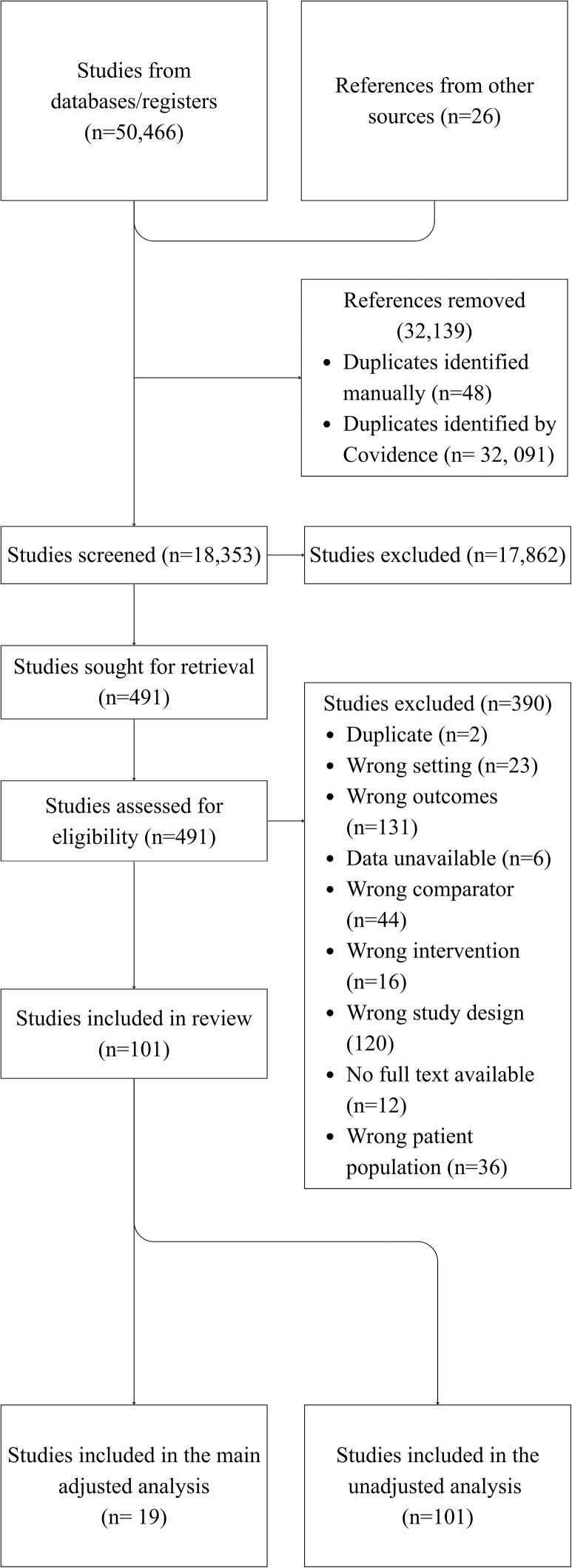
**PRISMA diagram of included studies**.

The search retrieved 18,353 studies after duplicates were removed. From these, 101 studies were eligible for inclusion, and included 92,915,856 women, 4,673,691 with GDM and 491,933 stillbirths. 19 studies were included in the adjusted results, encompassing 63,629,536 women, 3,375,630 with GDM and over 29,320 stillbirths. The process is summarised in the PRISMA Flow chart (Fig. 1). A summary of each included study is given in Supplementary Table S2.

Studies were carried out in over 40 countries across six continents. Only one study was from a low income country,^32^ 32 studies from middle income countries,^33^, ^34^, ^35^, ^36^, ^37^, ^38^, ^39^, ^40^, ^41^, ^42^, ^43^, ^44^, ^45^, ^46^, ^47^, ^48^, ^49^, ^50^, ^51^, ^52^, ^53^, ^54^, ^55^, ^56^, ^57^, ^58^, ^59^, ^60^, ^61^, ^62^, ^63^, ^64^, ^65^ and 68 from high income countries.^10^^,^^17^, ^66^, ^67^, ^68^, ^69^, ^70^, ^71^, ^72^, ^73^, ^74^, ^75^, ^76^, ^77^, ^78^, ^79^, ^80^, ^81^, ^82^, ^83^, ^84^, ^85^, ^86^, ^87^, ^88^, ^89^, ^90^, ^91^, ^92^, ^93^, ^94^, ^95^, ^96^, ^97^, ^98^, ^99^, ^100^, ^101^, ^102^, ^103^, ^104^, ^105^, ^106^, ^107^, ^108^, ^109^, ^110^, ^111^, ^112^, ^113^, ^114^, ^115^, ^116^, ^117^, ^118^, ^119^, ^120^, ^121^, ^122^, ^123^, ^124^, ^125^, ^126^, ^127^, ^128^, ^129^, ^130^, ^131^ One study included participants from multiple countries with different income levels including middle income countries and a high-income country^132^ although the high income country became so in 2013, 13 years after the study was conducted and therefore pragmatically, has been included in the analysis for middle income countries.

19 studies were prospective cohorts and 82 were retrospective cohorts (Supplementary Table S2). The oldest studies were published in 1992,^36^ and the most recent were published in 2025.^65^^,^^129^^,^^131^

Screening and diagnostic criteria for GDM varied significantly. Whilst many studies did not state the screening test used, when they did, the most commonly used screening tool was the 50 g oral glucose challenge test followed by the 75 g or 100 g oral glucose tolerance test. 51 studies adopted universal testing, 17 adopted risk-based testing while 33 gave no information about their screening methods (Supplementary Table S2). A variety of diagnostic methods were used including International Association of the Diabetes and Pregnancy Study Groups (IADPSG)^133^ and National Institute for Health and Care Excellence (NICE) guidelines.^134^ In high income countries, 31 used universal screening, 12 used risk-based screening and 25 were not stated. In middle income countries, 18 studies used universal screening, five used risk-based screening and nine did not state the method used. In the one study from a low income country, universal screening was used.

The quality of studies varied with 46 being poor quality overall, and 55 were good quality. Only 30 studies scored the maximum of two points for the ‘comparability’ domain in the Newcastle–Ottawa Scale due to controlling for at least two of the confounders: maternal age, BMI and smoking. However, most studies demonstrated characteristics of low bias in the Selection and Outcome domains.

**Fig. 2:**
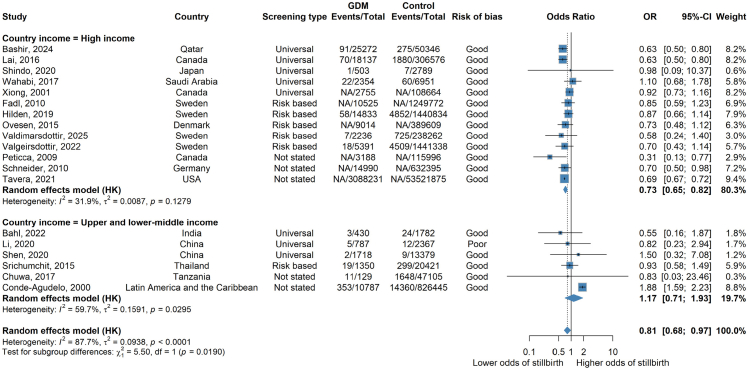
**Forest plot showing random effects meta-analysis of all adjusted cohort results overall and by country income level**. Forest plot of random effects meta-analysis of all adjusted results overall stratified by country income level. Where NA is stated, this refers to where a paper has not included the number of events/total number of events and only provides an adjusted odds ratio.

We found no clear evidence that a diagnosis of GDM was associated with increased risk of stillbirth overall, suggested by the pooled adjusted odds ratios (aOR) (aOR 0.81 [95% CI: 0.68–0.97; I^2^ = 87.7%] n = 19 studies) (Fig. 2). However, there was a high level of heterogeneity (I^2^ = 87.7%).

### Subgroup analysis by country income level

**Fig. 3:**
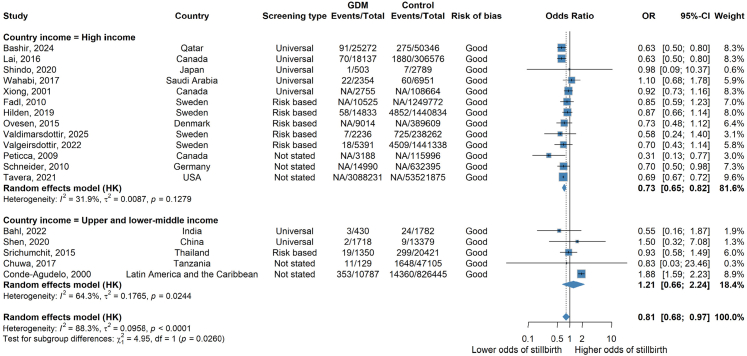
**Forest plot showing random effects meta-analysis of all adjusted cohort results overall and by country income level restricted to good quality studies**. Forest plot of random effects meta-analysis of adjusted results limited to only good quality studies. Where NA is stated, this refers to where a paper has not included the number of events/total number of events and only provides an adjusted odds ratio.

When cohort studies were stratified by country income level, there was evidence of a difference between adjusted estimates from high income countries and upper-and-lower middle income countries (Fig. 2). There were no adjusted estimates from low income countries. In high income countries, odds of stillbirth were (27%) lower in the GDM group compared to the control group (aOR 0.73 [95% CI: 0.65–0.82; I^2^ = 31.9%] n = 13 studies). There was moderate heterogeneity and all studies were of good quality, encompassing 62,702,836 women, 3,197,429 with GDM (Fig. 3). In the unadjusted results of high income countries only, a similar association was observed (OR 0.89 [95% CI: 0.77, 1.03 I^2^ = 91.8%] n = 69 studies) but with much higher heterogeneity (Supplementary Figure S1). Sensitivity analysis, removing studies that had one arm of zero events also showed a similar association (Supplementary Figure S2).

There was no clear evidence of an association between a diagnosis of GDM and stillbirth in upper and middle-income countries (aOR 1.17 [95% CI: 0.71–1.93; I^2^ = 59.7%] n = 6 studies) including 926,610 women, 15,201 with GDM and a total of 16,745 stillbirths (Fig. 2). When removing one poor quality study, the aOR was similar: (1.21 [95% CI: 0.66–2.24; I^2^ = 64.3%] n = 5 studies) (Fig. 3). The unadjusted results of all studies in middle income countries were directionally consistent but larger in magnitude (OR 2.12 [95% CI: 1.45–3.08; I^2^ = 66.2%] n = 32 studies) (Supplementary Figure S1). Sensitivity analysis, removing studies that had one arm of zero events also showed a similar association (Supplementary Figure S2).

### Subgroup analysis by country income level and type of GDM screening

**Fig. 4:**
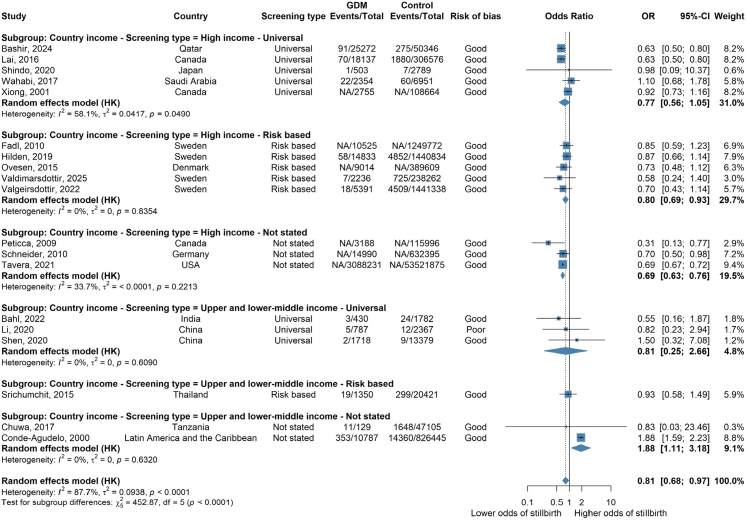
**Forest plot showing random effects meta-analysis of all adjusted cohort results overall and by country income level and type of GDM screening**. Forest plot of random effects meta-analysis of adjusted results overall stratified by country income level and GDM screening method (e.g. universal, risk based or not stated). Where NA is stated, this refers to where a paper has not included the number of events/total number of events and only provides an adjusted odds ratio.

When stratified by type of screening, the odds of stillbirth were lower when risk based screening was used in high income countries (aOR 0.80 [95% CI: 0.69–0.93; I^2^ = 0%] n = 5 studies) compared with universal screening (aOR 0.77 [95% CI: 0.56–0.1.05; I^2^ = 58.1%]; n = 5 studies) (Fig. 4). Though, when screening type was not reported, the odds of stillbirth were also lower (aOR 0.69 [95% CI: 0.63–0.76; I^2^ = 33.7%]; n = 3 studies). Within the upper and lower middle income countries there was were no differences detected when risk-based screening was used (aOR 0.93 [95% CI: 0.58–1.49] n = 1 study) compared to universal screening (aOR 0.81 [95% CI: 0.25–2.66; I^2^ = 0%] n = 3 studies) compared to when screening method was not stated (aOR 1.88 [95% CI: 1.11–3.18; I^2^ = 0%] n = 2 studies). However, it should be noted that the results in upper and lower middle income countries are based on few studies with small numbers of stillbirths and the I^2^ statistic suggested there was still unexplained between study heterogeneity.

The funnel plots for both adjusted results overall and adjusted results by country income level showed little evidence of small study effects, meaning the studies were evenly distributed and symmetrical around the average effect size. Overall for all adjusted results (Supplementary Figure S3), the Egger test suggested no evidence of funnel plot asymmetry (p-value = 0.295). When stratified by country income level, this was further reduced for high income countries. (Supplementary Figure S4, p-value = 0.42, n = 13 studies) whilst for upper and lower middle income countries visually there was no evidence of funnel plot asymmetry (Supplementary Figure S5) but there were only 6 studies (and 10 are required for the Egger test).

Meta-regression results showed that the association between GDM and stillbirth was significantly weaker in studies from high-income countries compared to those from upper- and lower-middle-income countries (Ratio of OR [ROR] = 0.52; 95% CI: 0.38–0.74; p = 0.0008). This suggests that the risk of stillbirth associated with GDM is substantially lower in high-income settings. Whilst there was no evidence of a difference in the association between GDM and stillbirth by type of screening (p = 0.9019). To explore this heterogeneity and potential moderators, we restricted by study quality and these estimates remained consistent.

## Discussion

Pooled analysis of adjusted estimates from cohort studies globally found no evidence of an increased risk of stillbirth in those with a GDM diagnosis compared to those without. However, in high income countries a diagnosis of GDM was associated with a 27% reduction in risk of stillbirth, whereas for upper and lower-middle income countries, an increased risk was found, but confidence intervals spanned the null value and there were few studies within this analysis. There were no studies in low-income countries that provided a confounder adjusted odds ratio. Our study found some evidence to suggest that risk based screening for GDM may be as useful as universal screening in high income settings.

Our study adds to the findings of a previous systematic review,^19^ that overall, the relationship between stillbirth and GDM globally remains unclear, though contrastingly, we did find that risk based screening may be as effective as universal screening in high income settings. Additionally, by taking only adjusted results, which better approximate the causal effect, and stratifying these by country income level, we have provided novel findings about the relationship between GDM and stillbirth in high income settings. This provides an opportunity to consider how to translate these results globally.

The increased risk of stillbirth with GDM has long been recognised^8^^,^^9^ making our finding of lower stillbirth rates in those with a diagnosis of GDM compared to those without in high-income countries initially surprising. However, when considering the management strategy outlined in national guidelines for GDM in high income countries including the UK, US, Australia and Canada, this is perhaps explained.^135^, ^134^, ^136^, ^137^ Women diagnosed with GDM in these settings receive extra antenatal appointments, ultrasound scans, dietary counselling, regular blood glucose monitoring and medications such as metformin and insulin to control blood glucose levels if diet control is insufficient. This means that additional risk factors such as faltering foetal growth can be identified and acted upon. In the UK, NICE guidelines advise delivery before 40 + 6 weeks gestation,^134^ The American College of Obstetricians and Gynaecologists (ACOG) advise delivery between 39 weeks and 40 + 6 weeks gestation for diet controlled and 39 to 39 + 6 weeks for well controlled GDM on medication.^138^ Birth at 39 weeks, reduces the length of exposure to the in-utero environment and therefore eliminates the rising, although still small, risk of stillbirth beyond this point. This intervention has been shown to reduce perinatal death even in the absence of GDM by the ARRIVE trial.^139^ Therefore, it is possible that the protective effect found in this study is at least partly due to the effect of these two main factors—timely birth and close antenatal monitoring.

In LMICs it is well recognised that barriers exist to accessing healthcare, often of poorer quality than in high-income settings. GDM is not always tested for or diagnosed in these settings, and when diagnosed, there are challenges in management due to the lack of availability of resources.^140^, ^141^, ^142^, ^143^, ^144^ As a result, there are likely to be more missed cases of GDM,^145^ restricted treatment options and suboptimal management even when diagnosed.^145^ It is possible that studies performed in low and middle income countries are including more women with little access to preventative healthcare outside of pregnancy, and so more of the cases classed as GDM may in fact be undiagnosed type 2 diabetes identified in pregnancy. Additionally, there is dramatic regional variation with a larger number of stillbirths occurring in Africa compared to South Asia and South America.^4^ The majority of low income countries are located within Africa, particularly Sub-Saharan Africa. Within this study, there were no low income countries included in the adjusted analyses and few studies from middle income countries, the majority of which are located in Asia.

The differences in diagnostic criteria are likely not due to different income settings alone; the glucose thresholds proposed by organisations such as the WHO, IADPSG,^138^ ACOG and NICE^134^, ^146^, ^147^ have changed, in some cases several times, over the years covered by studies in this review, and consensus regarding the best thresholds to use remains a controversial topic.^12^ Not only did GDM diagnostic criteria vary between studies, GDM screening practices also varied, further increasing heterogeneity. However, limited capacity exists for GDM screening in many LMICs,^141^ meaning that there are likely to be many more women with undiagnosed and unmanaged GDM. GDM is often not diagnosed until after 24 weeks of pregnancy, therefore early stillbirths prior to this point adds bias to the reporting of stillbirths which cannot be adjusted for by the gestational age of the stillbirth.

The definition of stillbirth also varies worldwide. The WHO defines stillbirth as the death of a baby antenatally, in labour or at birth ≥22 weeks or weighing ≥500 g, however for international comparisons this changes to ≥28 weeks or ≥1000 g. Amongst high-income countries this definition also varies widely, RCOG and NICE use a threshold of ≥24 weeks^9^ and in the USA, ACOG utilises ≥20 weeks and ≥350 g cut-off. Reaching an international consensus is unlikely to be achieved in the near future and uniformly counting stillbirths in LMICs remains difficult, making studies such as this challenging. Within this study, the definition of stillbirth was as per each author, as long as foetal death was spontaneous, and occurred after 20 weeks gestation but prior to birth.

The majority of studies in both high- and middle-income countries used universal screening, in line with WHO guidelines. Screening is a vital component in the management of GDM and therefore in the reduction of stillbirths. Overall, we did not find evidence that universal screening changed the risk of stillbirth when compared to risk-based screening globally, however risk based screening may be as effective as universal screening in high income countries. Though, with the current risk factors used for GDM screening, such as ethnicity,^147^ risk based screening is likely to be less useful in middle and lower income settings. Whilst the residual heterogeneity makes this interpretation challenging, the finding may be helpful when planning GDM screening programmes, as a risk based screening programme may support GDM diagnosis and be more cost effective and less labour intensive than universal screening, where resources may already be stretched.

However, in the UK, which uses risk based screening, cases of GDM are missed.^148^ Avalos et al. identified that 20% of cases of GDM would be missed when using NICE guidelines in a European population.^149^ When considering these guidelines in LMIC's, the existing criteria, encompassing ethnicity, would result in the majority of the population being considered high-risk. It also highlights that even in high income countries, with a relative wealth of resources, diagnostic processes fail to pick up all patients with GDM. This leads to missed opportunities for managing blood glucose, for extra scans to diagnose growth restriction or macrosomia, for counselling regarding shoulder dystocia and, ultimately, for mitigating the increased risk of stillbirth. GDM screening doesn't only play a role in reducing stillbirths, it is also vital in reducing maternal and neonatal morbidity and identifying women who may be at risk of type 2 diabetes in future. Further research is needed to help identify the ideal strategy taking into account cost, available resources and how to best identify the at-risk population.

Accounting for the actual uptake of screening should also be considered. Women who have declined screening can still have GDM but be accounted for in a non-GDM group. Bashir et al. describes a universal screening programme in Qatar where only 84.2% of women offered screening chose to receive it and Stacey et al. found that those who were at risk of GDM but did not receive screening had a 50% higher risk of stillbirth within some maternity units in the UK.^8^

We were only able to include 19/101 studies in the main analysis which adjusted for our pre-specified confounders, therefore our main conclusions are based on fewer studies. However, both adjusted and unadjusted results were reviewed and were similar. Notably, within upper and lower middle income countries, our main analyses are based on only six studies and the number of stillbirths remain low. The clinically relevant associations are limited as a result.

In the overall estimates the heterogeneity was relatively high, however, when stratifying by country income level the heterogeneity substantially reduced within both the high and upper and lower middle income groups. This showed that some of the heterogeneity can be explained by the fact that there are differences between settings. A moderate amount of heterogeneity does remain which may be explained by a combination of factors including varying stillbirth definitions and differing screening and diagnostic criteria for GDM, which in some studies changed multiple times. Additionally, factors such as BMI, glycaemic control during pregnancy and preexisting chronic diseases, study year and study quality may further confound these results. Subgroup analyses accounting for these factors was not in the scope of this review, and few studies provided data on glycaemic control during pregnancy or information regarding method of glycaemic control whether that be diet or medication. Though heterogeneity was high, this reduced significantly within the adjusted results when stratifying by country income level.

While subgroup analyses by country income level is a valuable approach, only one study was from a low income country, and did not provide an adjusted odds ratio, restricting the applications of the findings in this study.

We were only able to include observational studies in our analysis which carries a higher risk of bias. The studies included in the main analysis were cohort studies, only one of which was poor quality in the adjusted analyses. Within the unadjusted results, almost half of the studies were of poor quality, mainly due to their lack of adjusting for confounding variables. In contrast over half of the studies were of good quality and reassuringly, subgroup analysis of only high quality cohort studies correlated with the pooled results.

The lower risk of stillbirth with a diagnosis of GDM in high income countries should be interpreted cautiously. It may highlight the benefit of individualised approaches to diagnosis and management, along with differences in health system capacity, screening protocols and care pathways.

Women diagnosed with GDM in high income countries have a lower risk of stillbirth compared to those without a diagnosis of GDM. This is likely due to increased monitoring and antenatal care, as well as ensuring timely birth. In contrast, the risk in middle-income countries remains unclear and there is a lack of evidence from low-income countries. Implementing high-quality screening and GDM management programmes in middle and low-income settings is likely to reduce preventable stillbirths worldwide.

## Contributors

AM generated the idea for this work. AM, AF and GC planned the methodology. AM, AA and MT defined the search terms and completed the PROSPERO registration. VB, LS, AA, MT carried out the search strategy, screened abstracts and full texts. VB and LS completed the quality assessments. GC carried out the meta-analysis. VB, LS, AA, MT, GC & AM wrote the first draft of the manuscript. All other iterations of the manuscript were contributed to by all authors. The study data was accessed and verified by AM, GC, LS and VB.

## Data sharing statement

Data used for the systematic review is available upon contact with the corresponding author.

## Declaration of interests

We declare no competing interests.
