# Supplementary material for: Gestational diabetes and stillbirth: a systematic review and meta-analysis
Source: eClinicalMedicine. 2026 Jan 8;91:103751. doi: 10.1016/j.eclinm.2025.103751 (PMC12818070; doi:10.1016/j.eclinm.2025.103751)
Supplement: Supplementary File [file mmc1.docx]

**Gestational diabetes and stillbirth: a systematic review and meta-analysis**

Contents

[Supplementary Table 1: Newcastle-Ottawa Assessment for Cohort Studies 2](#_Toc216559612)

[Supplementary Table 2: Summary of Included Studies 13](#_Toc216559613)

[Supplementary Table 3: Studies excluded at full text review 41](#_Toc216559614)

[Supplementary figure 1: Random effects meta-analysis of unadjusted results from cohort studies by country income level 65](#_Toc216559615)

[Supplementary figure 2: Random effects meta-analysis of unadjusted results from cohort studies without continuity correction applied (sensitivity analysis) and therefore excluding studies with zero events in one or both arms 67](#_Toc216559616)

[Supplementary figure 3: Funnel plot of adjusted results from cohort studies 70](#_Toc216559617)

[Supplementary figure 4: Funnel plot of adjusted results from cohort studies restricted to high income countries 71](#_Toc216559618)

[Supplementary figure 5: Funnel plot of adjusted results from cohort studies restricted to upper and lower middle income countries 72](#_Toc216559619)

[Search strategy 73](#_Toc216559620)

[Reference list of included studies. 74](#_Toc216559621)

## Supplementary Table 1: Newcastle-Ottawa Assessment for Cohort Studies

|  | Selection | | | | Comparability | Outcome | | |
| --- | --- | --- | --- | --- | --- | --- | --- | --- |
| Included studies | Representativeness of the exposed cohort | Selection of non-exposed cohort | Ascertainment of exposure | Incident disease |  | Assessment of outcome | Length of follow-up | Adequacy of follow-up |
| Aberg, 2001 | ***** | ***** | ***** | ***** | ****** | ***** | ***** | ***** |
| Albolfazl, 2008 | ***** | ***** | ***** | ***** |  |  | ***** | ***** |
| Alfadhli, 2015 | ***** | ***** | ***** | ***** |  | ***** | ***** | ***** |
| Aljohani, 2008 | ***** | ***** | ***** | ***** |  | ***** | ***** | ***** |
| Al Khalaf, 2024 | ***** | ***** | ***** | ***** | ****** | ***** | ***** | ***** |
| Aviram, 2016 | ***** | ***** | ***** | ***** | ***** | ***** | ***** | ***** |
| Bahl, 2022 | ***** | ***** | ***** | ***** | ****** | ***** | ***** | ***** |
| Barakat, 2010 | ***** | ***** | ***** | ***** | ***** | ***** | ***** | ***** |
| Bashir, 2024 | ***** | ***** | ***** | ***** | ***** | ***** | ***** | ***** |
| Bahir, 2020 | ***** | ***** | ***** | ***** | ***** | ***** | ***** | ***** |
| Behuria, 2009 |  | ***** | ***** | ***** |  | ***** | ***** | ***** |
| Bukasa, 2023 | ***** | ***** | ***** | ***** | ***** | ***** | ***** | ***** |
| Berg, 2007 | ***** | ***** | ***** | ***** |  | ***** | ***** | ***** |
| Casey, 1997 | ***** | ***** | ***** | ***** |  | ***** | ***** | ***** |
| Chen, 2022 | ***** | ***** | ***** | ***** |  | ***** | ***** | ***** |
| Chirenje, 1992 | ***** | ***** | ***** | ***** |  | ***** | ***** | ***** |
| Chou, 2010 | ***** | ***** | ***** | ***** |  | ***** | ***** | ***** |
| Chung, 2022 |  | ***** | ***** | ***** | ****** | ***** | ***** | ***** |
| Chuwa, 2017 | ***** | ***** | ***** | ***** | ***** | ***** | ***** | ***** |
| Conde-Agudelo, 2000 | ***** | ***** | ***** | ***** | ****** | ***** | ***** | ***** |
| Darbandi, 2021 | ***** | ***** | ***** |  | ***** | ***** |  |  |
| Deitch, 2023 | ***** | ***** | ***** | ***** |  | ***** | ***** | ***** |
| Djelmis, 1997 |  |  |  | ***** |  |  |  | ***** |
| Donovan, 2017 | ***** | ***** | ***** | ***** | ****** | ***** | ***** | ***** |
| Ethridge, 2014 | ***** | ***** | ***** | ***** |  | ***** | ***** | ***** |
| El Mallah, 1997 | ***** | ***** | ***** | ***** |  | ***** | ***** | ***** |
| Fadl, 2010 | ***** | ***** | ***** | ***** | ****** | ***** | ***** | ***** |
| Feng, 2018 | ***** | ***** | ***** | ***** | ****** | ***** | ***** | ***** |
| Gardosi, 2013 | ***** | ***** | ***** | ***** | ***** | ***** | ***** | ***** |
| Goh, 2018 | ***** | ***** | ***** | ***** |  | ***** | ***** | ***** |
| Gortazar, 2021 | ***** | ***** | ***** | ***** | ****** | ***** | ***** | ***** |
| Gordon, 2013 | ***** | ***** | ***** | ***** | ****** | ***** | ***** | ***** |
| Goswami Mahanta, 2014 |  | ***** |  |  |  |  |  | ***** |
| Gunter, 2006 | ***** | ***** | ***** | ***** |  | ***** | ***** | ***** |
| Hilden, 2019 | ***** | ***** | ***** | ***** | ****** | ***** | ***** | ***** |
| Hossein-Nezhad, 2007 | ***** | ***** | ***** | ***** |  | ***** | ***** | ***** |
| Hucheon, 2013 | ***** | ***** | ***** | ***** |  | ***** | ***** | ***** |
| Ibiebele, 2016 | ***** | ***** | ***** | ***** | ***** | ***** | ***** | ***** |
| Jiang, 2017 | ***** | ***** | ***** | ***** |  | ***** | ***** | ***** |
| Johnstone 1990 | ***** | ***** | ***** | ***** |  | ***** | ***** | ***** |
| Jovonovic, 2015 | ***** | ***** | ***** | ***** |  | ***** | ***** | ***** |
| Kalra, 2013 |  | ***** | ***** | ***** |  | ***** | ***** | ***** |
| Karasneh, 2021 | ***** | ***** | ***** | ***** |  | ***** | ***** | ***** |
| Karen, 2024 | ***** | ***** | ***** | ***** | ***** | ***** | ***** | ***** |
| Karkia, 2023 | ***** | ***** | ***** | ***** |  | ***** | ***** | ***** |
| Karmon, 2009 | ***** | ***** | ***** | ***** |  | ***** | ***** | ***** |
| Kawakita, 2017 | ***** | ***** | ***** | ***** | ****** | ***** | ***** | ***** |
| Keshavarz, 2005 | ***** | ***** | ***** | ***** | ***** | ***** | ***** | ***** |
| Kleinwetcher, 2022 | ***** | ***** | ***** | ***** |  |  |  |  |
| Koning, 2018 | ***** | ***** | ***** | ***** |  | ***** | ***** | ***** |
| Kim, 2022 | ***** | ***** | ***** | ***** |  | ***** | ***** | ***** |
| Koukhan, 2020 | ***** | ***** | ***** | ***** |  | ***** | ***** | ***** |
| Kunzel, 2003 | ***** | ***** | ***** | ***** |  | ***** | ***** | ***** |
| Lai, 2016 | ***** | ***** | ***** | ***** | ***** | ***** | ***** | ***** |
| Lamminpaa, 2014 | ***** | ***** | ***** | ***** | ****** | ***** | ***** | ***** |
| Lamont, 2022 | ***** | ***** | ***** | ***** | ****** | ***** | ***** | ***** |
| Li 2020 | ***** | ***** | ***** | ***** |  | ***** | ***** | ***** |
| Magee, 1993 |  | ***** | ***** | ***** |  | ***** | ***** | ***** |
| Mahalakshmi, 2016 | ***** | ***** | ***** | ***** |  | ***** | ***** | ***** |
| Meghelli, 2019 | ***** | ***** | ***** | ***** |  | ***** | ***** | ***** |
| Morikawa, 2017 | ***** | ***** | ***** | ***** |  | ***** | ***** | ***** |
| Nava Guerrero | ***** | ***** | ***** | ***** |  | ***** | ***** | ***** |
| Nayak, 2013 | ***** | ***** | ***** | ***** |  | ***** | ***** |  |
| Nelson, 2024 | ***** | ***** | ***** | ***** |  | ***** | ***** | ***** |
| Nguyen, 2019 | ***** | ***** | ***** | ***** | ****** | ***** | ***** | ***** |
| Odar 2004 | ***** | ***** | ***** | ***** |  |  | ***** | ***** |
| Ohana, 2011 |  | ***** | ***** | ***** | ***** | ***** | ***** | ***** |
| Ovesen, 2015 | ***** | ***** | ***** | ***** | ****** | ***** | ***** | ***** |
| Ozumba, 2004 | ***** | ***** | ***** | ***** | ***** | ***** | ***** | ***** |
| Pan, 2015 | ***** | ***** | ***** | ***** | ****** | ***** | ***** | ***** |
| Panigrahi, 2020 | ***** | ***** | ***** | ***** |  |  | ***** | ***** |
| Peticca, 2009 | ***** | ***** | ***** | ***** | ****** | ***** | ***** |  |
| Pintaudi, 2014 | ***** | ***** | ***** | ***** | ***** | ***** | ***** | ***** |
| Ramachandran, 1998 | ***** | ***** |  | ***** | ****** |  | ***** | ***** |
| Reitzle, 2023 | ***** | ***** | ***** | ***** | ***** | ***** | ***** | ***** |
| Riskin, 2020 | ***** | ***** | ***** |  | ***** | ***** | ***** | ***** |
| Rosen, 2017 | ***** | ***** |  | ***** | ***** |  | ***** | ***** |
| Rosenstein, 2012 | ***** | ***** | ***** |  | ***** | ***** | ***** | ***** |
| Saha, 2014 |  | ***** | ***** |  |  |  | ***** | ***** |
| Schmidt, 2001 | ***** | ***** | ***** | ***** | ***** | ***** | ***** | ***** |
| Schneider, 2010 | ***** | ***** | ***** |  | ****** | ***** | ***** | ***** |
| Shand, 2008 | ***** | ***** | ***** | ***** | ****** | ***** | ***** | ***** |
| Sharipova, 2025 | ***** | ***** | ***** | ***** |  | ***** | ***** | ***** |
| She, 2021 |  | ***** |  |  |  |  | ***** | ***** |
| Shen, 2020 | ***** | ***** | ***** | ***** | ****** | ***** | ***** | ***** |
| Shindo, 2020 | ***** | ***** | ***** | ***** | ***** | ***** | ***** | ***** |
| Soliman, 2018 | ***** | ***** | ***** | ***** | ***** | ***** | ***** |  |
| Srichumchit, 2015 | ***** | ***** | ***** | ***** | ***** | ***** | ***** | ***** |
| Stone, 2002 |  |  |  | ***** | ***** | ***** | ***** |  |
| Svare, 2001 |  |  |  | ***** | ****** |  | ***** | ***** |
| Tanner, 2023 | ***** | ***** | ***** | ***** | ****** | ***** | ***** | ***** |
| Tavera, 2021 | ***** | ***** | ***** | ***** | ***** | ***** | ***** | ***** |
| Valdimarsdottir, 2024 | ***** | ***** | ***** | ***** | ****** | ***** | ***** | ***** |
| Valgeirsdottir, 2022 | ***** | ***** | ***** | ***** | ***** | ***** | ***** | ***** |
| Vivet-Lefebure 2007 | ***** | ***** |  | ***** | ***** | ***** | ***** | ***** |
| Wahabi, 2013 | ***** | ***** | ***** | ***** | ****** | ***** | ***** | ***** |
| Wahabi, 2017 | ***** | ***** |  | ***** | ****** |  | ***** |  |
| Winsloe, 2025 | ***** | ***** | ***** | ***** | ****** | ***** | ***** | ***** |
| Xiong, 2001 | ***** | ***** | ***** | ***** | ****** | ***** | ***** | ***** |
| Young, 2020 | ***** | ***** | ***** | ***** | ****** | ***** | ***** | ***** |
| Zahra, 2022 |  | ***** | ***** | ***** | ***** | ***** | ***** | ***** |

Methodology for completing the quality assessment for cohort studies:

This standardised form was used to complete the assessments for each study in duplicate. The total number of stars for each domain is recorded and the overall quality of the study is determined based on the having a particular number of stars in each domain.

**Selection**

**Representativeness of the exposed cohort**

| 1. Truly representative (one star) |
| --- |
| 1. Somewhat representative (one star) |
| 1. Selected group |
| 1. No description of the derivation of the cohort |

**Selection of the non-exposed cohort**

| 1. Drawn from the same community as the exposed cohort (one star) |
| --- |
| 1. Drawn from a different source |
| 1. No description of the derivation of the non exposed cohort |

**Ascertainment of exposure**

| 1. Secure record (e.g. surgical record) (one star) |
| --- |
| 1. Structured interview (one star) |
| 1. Written self report |
| 1. No description |
| 1. Other |

**Demonstration that outcome of interest was not present at start of study (basically is the study period long enough e.g. for GDM like at least 9 months)**

| 1. Yes (one star) |
| --- |
| 1. No |

**Comparability**

**Comparability of the cohorts on the basis of the design or analysis controlled for cofounders**

| 1. The study controls for at least 2 of age, BMI and smoking (2 stars) |
| --- |
| 1. The study controls for other risk factors (please list) (one star) |
| 1. Cohorts are not comparable on the basis of the design or analysis controlled for confounders |

**Outcome**

**Assessment of outcome**

| 1. Independent blind assessment (one star) |
| --- |
| 1. Record linkage (one star) |
| 1. Self report |
| 1. No description |
| 1. Other |

**Was follow-up long enough for outcomes to occur- please indicate the length of follow up and rationale behind your assessment**

| 1. Yes (one star) |
| --- |
| 1. No |

**Adequacy of follow-up of cohorts**

| 1. Complete follow-up. All subjects accounted for (one star) |
| --- |
| 1. Subjects lost to follow up unlikely to introduce bias- numbers lost less than or equal to 20% or description of those lost to follow up suggested no different from those followed. (one star) |
| 1. Follow up rate less than 80% with no description of those lost |
| 1. No statement |

**Final Assessment:**

| Good quality:  3 or 4 stars in selection domain AND 1 or 2 stars in comparability domain AND 2 or 3 stars in outcome/exposure domain |
| --- |
| Fair quality:  2 stars in selection domain AND 1 or 2 stars in comparability domain AND 2 or 3 stars in outcome/exposure domain |
| Poor quality:  0 or 1 star in selection domain OR 0 stars in comparability domain OR 0 or 1 stars in outcome/exposure domain |

## Supplementary Table 2: Summary of Included Studies

| **First Author** | **Year** | **Country** | **Study Design** | **Universal or risk-based testing** | **Screening Test** | **Diagnostic criteria for GDM** | **Stillbirth definition by gestation** | **Country Income Level** | **Confounders adjusted for** |
| --- | --- | --- | --- | --- | --- | --- | --- | --- | --- |
| Aberg (1) | 2001 | Sweden | Retrospective Cohort | Universal | 75g OGTT at 27-28 weeks gestation | 2hr ≥ 9 mmol/L | Not stated | High | None |
| Abolfazl (2) | 2008 | Iran | Retrospective cohort | Not stated | Not stated | Not stated | Not stated | Upper Middle | None |
| Al Khalaf (3) | 2024 | Sweden | Retrospective cohort | Not stated | Not stated | Not stated | 22 weeks | High | Maternal age, body mass index, smoking, education, country of origin, year of delivery and comorbidities (cardiovascular disease, diabetes, gestational diabetes, hypertensive disorders during pregnancy) |
| Alfadhli (4) | 2015 | Saudi Arabia | Prospective Cohort | Universal | FPG at booking +/- 75g OGTT at 24-28 weeks | FPG 5.1-7.0 OGTT:  Fasting ≥5.1 mmol/L  1hr ≥10 mmol/L  2hr ≥8.5mmol/L | 22 weeks | High | Not stated |
| Aljohani (5) | 2008 | Canada | Retrospective Cohort | Universal | 1992- 1997: 50g OGCT at 24 weeks + 100g OGTT  1998 – 2004:  50g OGCT + 75g OGTT | Not stated | 20 weeks | High | None |
| Aviram (6) | 2016 | Israel | Retrospective cohort | Universal | 50g OGCT +/- 100g OGTT at 24-28 weeks | Fasting ≥95 mg /dL  1hr ≥180 mg /dL  2hr ≥155 mg /dL  3 hr ≥140 mg | 34 weeks | High | Not stated |
| Bahl (7) | 2022 | India | Retrospective cohort | Universal | 75g OGTT in first trimester +/- 24-28 weeks +/- 34-36 weeks | 2hr >140 mg/dL | 28 weeks | Lower middle | Maternal age, heights, years at school, early pregnancy BMI, religion, type of family, family wealth |
| Barakat (8) | 2010 | Oman | Retrospective cohort | Universal | 75g OGTT at 24-28 weeks | Not stated | Not stated | High | BMI, hypertension |
| Bashir (9) | 2020 | Qatar | Retrospective Cohort | Universal | Fasting blood glucose at first antenatal visit +/- 75g OGTT at 16 and 24 weeks for high risk patients or 24 weeks for low risk patients | FBG ≥ 5.1 mmol/l, 1 hr ≥ 10.0 mmol/l  2 hr ≥ 8.5 mmol/l | Not stated | High | Age, pre-pregnancy weight, and  weekly gestational weight gain |
| Bashir (10) | 2024 | Qatar | Retrospective cohort | Universal | Fasting blood glucose at first antenatal visit +/- 75g OGTT at 16 and 24 weeks for high risk patients or 24 weeks for low risk patients | FBG 5.1 – 6.9 mmol/l  1 hr ≥ 10.0 mmol/l  2 hr 8.5 – 11.0 mmol/l | Not stated | High | Age |
| Behuria (11) | 2009 | India | Prospective cohort | Universal | 50g OGCT at 24-28/40 +/- OGTT | Not stated | Not stated | Lower Middle | None |
| Berg (12) | 2007 | Sweden | Retrospective cohort | Universal | RPG +/-  75g OGTT | RPG ≥11.1  2 hr OGTT ≥7.8mmol/l | Not stated | High | None |
| Bukasa (13) | 2023 | United Kingdom | Retrospective cohort | Risk based | OGTT | Fasting ≥ 5.6 mmol/L  2 hr ≥ 7.8 mmol/L | 24 weeks | High | Age, parity, ART |
| Casey (14) | 1997 | USA | Retrospective Cohort | Risk based | 50g OGCT immediately or at 24-28 +/- 100g OGTT | FPB ≥ 105 mg/dl  1hr ≥ 190 mg/dl  2hr ≥ 165 mg/dl  3hr ≥ 145 mg/dl | Not stated | High | None |
| Chen (15) | 2022 | China | Retrospective cohort | Universal | 75g OGTT at 24-28 weeks | FBG  ≥ 5.1 mmol/l  1 hr  ≥ 10.0 mmol/l  2 hr  ≥  8.5 mmol/l | 20 weeks | Upper middle | None |
| Chirenje (16) | 1992 | Zimbabwe | Retrospective cohort | Risk based | 50 g OGCT at booking +/- 75g OGTT | FBG ≥6.0 mmol/l  2 hr ≥7.0 mmol/l | Not stated | Lower Middle | None |
| Chou (17) | 2010 | Taiwan | Retrospective Cohort | Universal | 50g OGCT immediately or at 24-28 weeks followed by 100g OGTT if serum glucose at 1 hr ≥140 mg/dL | Fasting ≥95 mg /dL  1hr ≥180 mg /dL  2hr ≥155 mg /dL  3 hr ≥140 mg /dL  OR  Fasting ≥105 mg /dL  1hr ≥190 mg /dL  2hr ≥165 mg /dL  3 hr ≥145 mg /dL | Not stated | High | None |
| Chung (18) | 2022 | South Korea | Retrospective cohort | Universal | 50g OGCT at 24-28 weeks +/- 100g OGTT | Fasting ≥95 mg /dL  1hr ≥180 mg /dL  2hr ≥155 mg /dL  3 hr ≥140 mg /dL | Not stated | High | Maternal age and pre-pregnancy BMI. |
| Chuwa (19) | 2017 | Tanzania | Retrospective cohort | Not stated | Not stated | Not stated | 28 weeks | Lower middle | Maternal age, PIH, number of antenatal clinic visits, alcohol consumption during pregnancy, placental abruption, pre-eclampsia, PROM, anaemia |
| Conde-Agudelo (20) | 2000 | Uruguay, Argentina, Peru, Colombia, Honduras, Paraguay, El Salvador, Chile, Bolivia, Costa Rica, Panama, Dominican Republic, Nicaragua, Brazil, Ecuador, Mexico, Bahamas and Venezuela | Retrospective cohort | Not stated | Not stated | Not stated | 20 weeks | Lower middle, Upper Middle and High | Maternal age, BMI, smoking, diseases during pregnancy, geographic area, parity, interpregnancy interval, marital status, education |
| Darbandi (21) | 2021 | Iran | Retrospective Cohort | Universal | 50g OGCT +/- 1100g OGTT | Fasting ≥95 mg /dL  1hr ≥180 mg /dL  2hr ≥155 mg /dL  3 hr ≥140 mg /dL | 22 weeks | Upper Middle | Age, place of residence, ethnicity, physical activity |
| Deitch (22) | 2023 | Australia | Retrospective cohort | Universal | 75g OGTT at 24-28 weeks | FPG < 5.1 mmol/L  2 hr < 6.8 mmol/L | 20 weeks | High | Not stated |
| Djelmis (23) | 1997 | Czechia | Retrospective Cohort | Universal | 75g OGTT at 20-28 weeks | Fasting ≥7.8 mmol/L  2hr ≥ 11.1 mmol/L. | 28 weeks | High | None |
| Donovan (24) | 2017 | Canada | Retrospective Cohort | Universal | 50g OGCT at 24-28 weeks, +/- 75g OGTT | OGCT >10.3mmol/L  OGTT –  Fasting ≥5.1 mmol/L  1 hr 10-10.5 mmol/L  2 hr ≥ 8.5 mmol/L  OR  Fasting ≥5.3 mmol/L  1 hr ≥10.6 mmol/L  2 hr ≥ 9 mmol/L | 20 weeks | High | Maternal weight, pre-pregnancy medical conditions (heart disease, hypertension, chronic renal disease, epilepsy, asthma, SLE, Crohn’s disease), smoking during pregnancy, maternal urban/rural residence, maternal median household income |
| El Mallah (25) | 1997 | Saudi Arabia | Retrospective Cohort | Universal | 50g OGCT at 24-28 weeks +/- 100g OGTT | Fasting ≥ 5.8 mmol/L  1 hr ≥10.6 mmol/L  2 hr ≥ 9/2 mmol/L  3 hr ≥ 8.1 mmol/L | Not stated | High | None |
| Ethridge (26) | 2014 | USA | Retrospective Cohort | Not stated | 50g OGCT or 100g OGTT | Fasting ≥95 mg /dL  1hr ≥180 mg /dL  2hr ≥155 mg /dL  3 hr ≥140 mg /dL  Or  Fasting ≥92 mg /dL  1hr ≥180 mg /dL  2hr ≥153 mg /dL | Not stated | High | None |
| Fadl (27) | 2010 | Sweden | Retrospective Cohort | Risk based | Repeated random capillary blood glucose  Small area of Sweden using 75g OGTT | Fasting ≥ 6.1 mmol/L  2 hr ≥ 9.0 mmol/L | 28 weeks | High | Maternal age, BMI, CHD, ethnicity, parity, smoking |
| Feng (28) | 2018 | China | Retrospective Cohort | Not stated | 75g OGTT at 24-24 weeks | Fasting ≥ 5.1 mmol/L  1 hr ≥ 10.0 mmol/L  2 hr 8.5 mmol/L | 20 – 28 weeks | Upper Middle | Age, BMI, primparity, anaemia |
| Gardosi (29) | 2013 | United Kingdom | Retrospective cohort | Not stated | Not stated | Not stated | 24 weeks | High | Maternal characteristics |
| Goh (30) | 2018 | Malaysia | Retrospective Cohort | Not stated | Not stated | Not stated | 24 weeks | Upper Middle | None |
| Gordon (31) | 2013 | Australia | Retrospective Cohort | Not stated | Not stated | Not stated | 22 weeks | High | Aboriginal, age, parity, pre-existing hypertension, smoking sex of child, pre-eclampsia, area of residence |
| Gortazar (32) | 2021 | Spain | Retrospective cohort | Universal | 50g OGCT +/- 100g OGTT | Not stated | 22 weeks | High | Maternal age, chronic hypertension, dyslipidaemia, year of delivery, smoking status |
| Goswami Mahanta (33) | 2014 | India | Prospective cohort | Universal | 75g OGTT | 2 hr 140-199 mg/dl | Not stated | Lower middle | None |
| Gunter (34) | 2006 | Germany | Retrospective cohort | Not stated | Not stated | Not stated | Not stated | High | None |
| Hilden (35) | 2019 | Sweden | Prospective Cohort | Risk based | RPG in first trimester +/- 75g OGTT | FBG ≥ 7.0 mmol/l  2 hr ≥10.0 mmol/l | 1998-2007: 28 weeks  2008- 2012:  22 weeks | High | Maternal age, smoking, BMI, non-Nordic origin, parity, chronic hypertension. |
| Hossein-Nezhad (36) | 2007 | Iran | Prospective Cohort | Universal | 50g OGCT at 24-28 weeks. +/- 100g OGTT | Fasting ≥95 mg /dL  1hr ≥180 mg /dL  2hr ≥155 mg /dL  3 hr ≥140 mg /dL | Not stated | Upper Middle | None |
| Hutcheon (37) | 2013 | USA | Retrospective Cohort | Not stated | Not stated | Not stated | 20-28 weeks | High | None |
| Ibiebele (38) | 2016 | Australia | Retrospective Cohort | Not stated | Not stated | Not stated | 20 weeks or 400g | High | Age, marital status, socioeconomic status, geographic location |
| Jiang (39) | 2017 | Australia | Retrospective Cohort | Universal | 50g OGCT at 24-28 weeks+/- 75g OGTT | Fasting ≥5.5 mmol/l  2 hr ≥8.0 mmol/l  OR  Fasting ≥ 5.1 mmol/l  1 hr ≥10.0 mmol/l  2 hr ≥8.5 mmol/l | Not stated | High | None |
| Johnstone (40) | 1990 | Kuwait | Retrospective cohort | Not stated | Not stated | Fasting ≥7.0 mmol/l  2 hrs ≥10.0 mmol/l | Not defined | High | None |
| Jovanovic (41) | 2015 | USA | Retrospective Cohort | Not stated | Not stated | Not stated | 24 weeks | High | None |
| Kalra (42) | 2013 | India | Prospective Cohort | Universal | 75g OGTT at 24-28 weeks | 2hr ≥140 mg/dL | Not stated | Lower Middle | None |
| Karasneh (43) | 2023 | Jordan | Retrospective cohort | Not stated | Not stated | Not stated | Not defined but all women recruited after 20 weeks | Lower Middle | None |
| Karen (44) | 2024 | Israel | Retrospective cohort | Not stated | Not stated | Not stated | Not stated | High | Maternal age, parity, previous cesarean delivery, neonatal weight, and induction of labour |
| Karkia (45) | 2023 | United Kingdom | Prospective cohort | Risk based | 75g OGTT in first trimester +/- 75g at 24-28 weeks | Fasting ≥ 5.6 mmol/L  2 hr ≥ 7.8 mmol/L | 24 weeks | High | Not stated |
| Karmon (46) | 2009 | Israel | Retrospective Cohort | Universal | 50g OGCT at 24-28 weeks. +/- 100g OGTT | Fasting ≥95 mg /dL  1hr ≥180 mg /dL  2hr ≥155 mg /dL  3 hr ≥140 mg /dL | Not defined | High | None |
| Kawakita (47) | 2017 | USA | Retrospective cohort | Not stated | Not stated | Not stated | Not stated | High | Adjusted for confounding variables but did not state OR for GDM |
| Keshavarz (48) | 2005 | Iran | Prospective Cohort | Risk based | 50g OGCT at booking or 24-28 weeks +/- 100g OGTT | Fasting ≥95 mg /dL  1hr ≥180 mg /dL  2hr ≥155 mg /dL  3 hr ≥140 mg /dL | Not stated | Upper Middle | None |
| Kim (49) | 2022 | South Korea | Retrospective cohort | Not stated | 50g OGTT | 100-g  OGTT between 24 and 28 weeks of gestation Before December 2005  (period 1), women with 50-g OGTT level≥140 mg/dl  (7.8 mmol/L) underwent 100-g OGTT with GDM diagnosed when the following two or more of plasma glucose  levels were above the NDDG criteria: fasting≥105 mg/  dl (5.8 mmol/L), 1-h≥190 mg/dl (10.6 mmol/L),  2-h≥165 mg/dl (9.2 mmol/L), and 3-h≥145 mg/dl  (8.0 mmol/L). After December 2005 (period 2), the cut off criteria (≥140 mg/dl (7.8 mmol/L) in low-risk women  and≥130 mg/dl (7.2 mmol/L) (in high-risk women)  of the 50-g OGTT were used | Not stated but excluded IUFD before 24 weeks | High | None |
| Kleinwetcher (50) | 2022 | Germany | Prospective cohort | Universal | 50g 1 Hour OGCT, then 2 hour 75g OGTT | Fasting: 92mg/dL; 1 hour: 180 mg/dL; and 2 hours: 153 mg/dL. | 24 weeks | High | BMI, week of gestation at the onset of COVID-19 symptoms (or at PCR-positive test result), multiples, maternal age at the time of positive COVID-19 test result, language competence (communication possible with or without problems), nicotine or smoking during pregnancy (yes or no), parity (0 or ≥1), and hypertensive disorders in pregnancy (yes or no). |
| Koning (51) | 2018 | Netherlands | Retrospective Cohort | Risk based | 75g OGTT at 24-28 weeks | Fasting ≥7.0 mmol/l  2 hr ≥7.8 mmol/l | Not stated | High | None |
| Kouhkan (52) | 2020 | Iran | Retrospective Cohort | Not stated | Not stated | 75g 2 hr OGTT at 24-28 weeks  0-hr (fasting) ≥ 92 mg/dL; 1-hr ≥ 180 mg/dL; or 2- hr ≥ 153 mg/dL. | Not stated | Upper Middle | Adjusted for maternal characteristics |
| Kunzel (53) | 2003 | Germany | Retrospective Cohort | Not stated | Not stated | Not stated | Not stated | High | None |
| Lai (54) | 2016 | Canada | Retrospective Cohort | Universal | 50g OGCT at 24-28 weeks. +/- 75-g OGTT | Fasting  ≥5.3 mmol/L 1 h ≥ 10.6 mmol/L  2 h ≥ 8.9 mmol/L | 20 weeks | High | Maternal characteristics, mode of delivery |
| Lamminpaa (55) | 2014 | Finland | Retrospective Cohort | Universal | 75g OGTT at 24-28 weeks | Fasting  ≥5.3 mmol/L 1 h ≥ 10.0 mmol/L  2 h ≥ 8.6 mmol/L | Not stated | High | Smoking, anemia, placenta  previa, In vitro fertilisation (IVF), fertility treatment other  than IVF, previous caesarean section, and hospitalization  because of bleeding, pre-pregnancy BMI ≥25 and  preeclampsia |
| Lamont (56) | 2022 | Finland, Malta, Scotland | Retrospective Cohort | Not stated | Not stated | Not stated | 22 weeks (Finland and Malta)  24 weeks (Scotland) | High | First pregnancy outcome, maternal age, BMI, marital status, smoking status, socioeconomic status, pre-existing hypertension, pre-eclampsia, placental abruption, placenta praevia, antepartum haemorrhage, fetal growth restriction, gestational age at birth |
| Li (57) | 2020 | China | Retrospective Cohort | Universal | 75g OGTT at 24-28 weeks | Fasting ≥5.1 mmol/l  1 hr ≥ 10.0 mmol/l  2 hr ≥ 8.5 mmol/l | 28 weeks | Upper Middle | None |
| Magee (58) | 1993 | USA | Prospective Cohort | Universal | 50g OGCT at 28 weeks +/- 100g OGTT | Fasting ≥5.9 mmol/l  1 hr ≥10.6 mmol/l  2 hr ≥9.2 mmol/l  3 hr ≥8.1 mmol/l  OR  Fasting ≥5.3 mmol/l  1 hr ≥10.1 mmol/l  2 hr ≥8.7 mmol/l  3 hr ≥7.8 mmol/l | 24 weeks | High | None |
| Mahalakshmi (59) | 2016 | India | Retrospective Cohort | Universal | OGTT at 24-28 weeks | Fasting ≥95 mg /dL  1hr ≥180 mg /dL  2hr ≥155 mg /dL  3 hr ≥140 mg /dL  OR  Fasting ≥105 mg /dL  1hr ≥190 mg /dL  2hr ≥165 mg /dL  3 hr ≥145 mg /dL | Not stated | Lower Middle | None |
| Meghelli (60) | 2019 | France | Retrospective cohort | Not stated | 1999 – 2009: 50g OGCT +/- 100g OGTT  2009- 2014:  75g OGTT | 1999- 2009:  Fasting ≥ 5.3 mmol/L  1 hr ≥ 10.1 mmol/L  2 hr ≥ 8.7 mmol/L  3 hr ≥ 7.8 mmol/L  2009- 2014:  Fasting ≥5.1 mmol/l  1 hr ≥ 10.0 mmol/l  2 hr ≥ 8.5 mmol/l | Not defined | High | Not stated |
| Morikawa (61) | 2017 | Japan | Retrospective Cohort | Universal | 75g OGTT | Fasting ≥ 92 mg/dl  1 hr ≥180 mg/dl  2 hr ≥153 mg/dl | 22 weeks | High | None |
| NavaGuerrero (62) | 2021 | Mexico | Retrospective cohort | Universal | 75g 2 hour OGTT | Two or more altered values regarding serum glucose; fasting,≥95 mg/dl, 1 h≥180 mg/dl, and 2 h≥155 mg/dl | Not stated | Upper middle | None |
| Nayak (63) | 2013 | India | Prospective Cohort | Universal | 75g OGTT at 24-32 weeks | Fasting ≥ 5.1 mmol/l  1 hr ≥10 mmol/l  2 hr ≥8.5 mmol/l | Not stated | Lower Middle | None |
| Nelson (64) | 2024 | Canada | Retrospective cohort | Universal | 75g OGTT | FPG ≥ 5.1 mmol/L  1 hr ≥ 10.0 mmol/L  2 hr ≥ 8.5 mmol/L | Not stated | High | None |
| Nguyen (65) | 2019 | Vietnam | Prospective Cohort | Universal | 75g OGTT between 24-28 weeks | Fasting ≥ 5.3 mmol/l  1 hr ≥ 10.0 mmol/l  2 hr ≥ 8.6 mmol/l  OR  Fasting ≥ 6.0 mmol/l  2 hr ≥ 9.0 mmol/l  OR  Fasting ≥ 5.1 mmol/l  1 hr ≥ 10.0 mmol/l  2 hr ≥ 8.5 mmol/l  OR  Fasting ≥ 5.6 mmol/l  2 hr ≥ 7.8 mmol/l | 28 weeks | Lower Middle | Maternal age, education, pre-pregnancy BMI, parity, passive smoking, alcohol drinking, previous GDM, macrosomia, preterm birth, caesarean birth, family history of diabetes or hypertension, infant sex |
| Odar (66) | 2004 | Uganda | Prospective Cohort | Universal | 75g OGTT | 2 hr ≥140 mg/dl | Not stated | Low | None |
| Ohana (67) | 2011 | Israel | Retrospective Cohort | Not stated | Not stated | Not stated | 22 weeks | High | Not stated |
| Ovesen (68) | 2015 | Denmark | Retrospective Cohort | Risk based | 75g OGTT at 27-30 weeks | 2 hr ≥ 9 mmol/L | Greater than 22 weeks | High | Maternal age, parity, smoking during pregnancy, gestational age, birth weight, BMI, gender of fetus, calendar year |
| Ozumba (69) | 2004 | Nigeria | Retrospective cohort | Risk based | 75g OGTT | Fasting ≥ 7 mmol/l  2 hr ≥ 11.1 mmol/l | Not stated | Lower Middle | None |
| Pan (70) | 2015 | China | Retrospective cohort | Universal | 50g OGCT at 24-28 weeks +/- 75g OGTT | Fasting ≥ 5.1 mmol/l  1 hr ≥ 10.0 mmol/l  2 hr ≥ 8.5 mmol/l  OR  FPG ≥7.0 mmol/l and/or 2 h PG ≥11.1 mmol/l  OR FPG ≥6.1 mmol/l but <7.0 mmol/l and 2hr <7.8 mmol/l | Not stated | Upper Middle | None |
| Panigrahi (71) | 2020 | India | Prospective Cohort | Universal | 75g OGTT between 24-32 weeks | 2 hr ≥140 mg/dL | Delivery of a dead baby at or  after 22 weeks of gestation | Lower Middle | None |
| Peticca (72) | 2009 | Canada | Retrospective Cohort | Not stated | Not stated | Not stated | 20 weeks | High | Maternal age, BMI, multiple births, smoking in pregnancy, parity, use f assisted reproductive technology, first trimester visit, antenatal care provider |
| Pintaudi (73) | 2015 | Italy | Retrospective cohort | Risk based | At booking if high risk or at 24-28 weeks | Fasting ≥92 mg /dL  1hr ≥180 mg /dL  2hr ≥153 mg /dL | Not stated | High | None |
| Ramachandran (74) | 1998 | India | Prospective Cohort | Universal | 75g OGTT at 24-28 weeks, +/- 100g OGTT | Fasting ≥140 mg/dL  2 hr ≥ 200 mg/dl | Not stated | Lower Middle | Age, BMI |
| Reitzle (75) | 2023 | Germany | Retrospective cohort | Not stated | Not stated | Not stated | 24 weeks | High | None |
| Riskin (76) | 2020 | Israel | Retrospective cohort | Not stated | Not stated | Not stated | Not defined | High | None |
| Rosen (77) | 2017 | Israel | Retrospective cohort | Not stated | 50g OGCT +/- 100g OGTT | Fasting ≥ 105 mg/dL  1 hr ≥ 190 mg/dL  2 hr ≥ 165 mg/dL  3 hr ≥ 145 mg/dL | Not defined | High | Maternal age, parity, previous caesarean, presence of hypertensve disorders, amniotic fluid imbalance, gestational age at delivery, LGA/AGA |
| Rosenstein (78) | 2012 | USA | Retrospective cohort | Universal | 50g OGCT +/- 100g OGTT | Not stated | 36 weeks | High | None |
| Saha (79) | 2014 | India | Retropective cohort | Universal | 50g OGCT for between 24-28 weeks +/- OGTT | FPG >126 mg/dl  RPG > 200 mg/dl | Not stated | Lower Middle | None |
| Schmidt (80) | 2001 | Brazil | Prospective Cohort | Universal | 75g OGTT between 24-28 weeks | FPG ≥ 5.3mmol/l  1 hr ≥ 10mmol/l  2hr ≥ 8.6mmol/l  OR  FPG ≥ 7 mmol/l  2hr ≥ 7.8 mmol/l | 28 weeks | Upper Middle | None |
| Schneider (81) | 2011 | Germany | Retrospective cohort | Not stated | Not stated | Not stated | Newborn with no signs of life and a birthweight of at least 500g | High | Maternal age, smoking, BMI, parity, weight gain in pregnancy, nationality, employment status, multiple pregnancy |
| Shand (82) | 2008 | Australia | Retrospective cohort | Universal | 50g OGCT at 26-28 weeks +/- 75g OGTT | FPG ≥ 5.5 mmol/l  2 hr ≥ 8.0 mmol/l | 30 weeks | High | None |
| Sharipova (83) | 2025 | Kazakhstan | Retrospective cohort | Not stated | Not stated | Not stated | Not stated | Upper middle | None |
| She (84) | 2021 | China | Prospective cohort | Not stated | Not stated | Not stated | Not stated | Upper middle | None |
| Shen (85) | 2020 | China | Retrospective cohort | Universal | 75g OGTT at 26-28 weeks | Fasting ≥ 5.1 mmol/L  1 hr ≥ 10 mmol/L  2 hr ≥ 8.5 mmol/L | 20 weeks | Upper Middle | Pre-delivery BMI, gestational hypertension, maternal-age |
| Shindo (86) | 2020 | Japan | Retrospective Cohort | Universal | 75g OGTT before 20 weeks if risk factors present  50 OGCT from 24-28 weeks if no risk factors present +/- 75g OGTT | Fasting ≥ 5.1 mmol/L  1 hr ≥ 10 mmol/L  2 hr ≥ 8.5 mmol/L | Not stated | High | Maternal age, height, pre-pregnancy weight, multiparous |
| Soliman (87) | 2018 | Qatar | Retrospective Cohort | Not stated | 75g OGTT | Fasting ≥ 5.1 mmol/L  1 hr ≥ 10 mmol/L  2 hr ≥ 8.5 mmol/L | Not defined | High | None |
| Srichumchit (88) | 2015 | Thailand | Retrospective Cohort | Risk based | 50g OGCT at 24-28 weeks +/- 100g OGTT | Fasting ≥ 105 mg/dL  1 hr ≥ 190 mg/dL  2 hr ≥ 165 mg/dL  3 hr ≥ 145 mg/dL | Not defined | Upper Middle | None |
| Stone (89) | 2002 | Australia | Retrospective Cohort | Not stated | Not stated | Not stated | Not stated | High | Maternal country of birth, aboriginality,  marital status, age, gestation, parity, socioeconomic status based on postcode, previous perinatal death,  congenital malformation in current pregnancy, macrosomia, hypertension or pre-eclampsia, sex of the child and the birth condition |
| Svare (90) | 2001 | Denmark | Prospective Cohort | Risk based | 1996-1998: FBG +/- 75g OGTT +/- repeat at 30-32 weeks 1998-2000: 75g OGTT +/- repeat at 30-32 weeks | Fasting > 5.9 mmol/l  1 hr > 12.0 mmol/l  2hr > 8.9 mmol/l | 28 weeks | High | None |
| Tanner (91) | 2023 | USA | Retrospective Cohort | Not stated | Not stated | Not stated | 28 weeks | High | Maternal age, paternal age, maternal education, maternal race, maternal Hispanic origin, maternal nativity, number of previous livebirths, previous caesarean, infertility treatment, hypertension, maternal smoking, maternal pre-pregnancy BMI, timing of prenatal care, infant sex, supplemental nutrition |
| Tavera (92) | 2021 | USA | Retrospective Cohort | Not stated | Not stated | Not stated | Not defined | High | Age group, race/ethnicity, household income, insurance status, admission day, hospital census region, hospital size and teaching status |
| Valdimarsdottir (93) | 2025 | Sweden | Retrospective cohort | Risk based | 75g 2 hour OGTT | Fasting venous plasma ≥7.0 mmol/L and a 2 h cut-off value after OGTT ≥10.0 mmol/L | 28 weeks (Up to July 2008) then 22 weeks thereafter. | High | Adjusted for maternal age, parity, education, country of birth and BMI. |
| Valgeirsdottir (94) | 2022 | Sweden | Retrospective | Risk based | 75g 2 hour OGTT | Fasting glucose was ≥7.0 mmol/l and/or two-hour  cut-off values were either ≥8.9 mmol/L, ≥10.0 mmol/L or ≥12.2 mmol/L | Not stated | High | Adjusted for maternal age, body mass index, country of birth, chronic hypertensive disease, smoking and parity |
| Vivet-Lefebure (95) | 2007 | Reunion Island, France | Retrospective Cohort | Universal | 100g OGTT at booking or 24-28 weeks | Fasting ≥95 mg/dl  1 hr ≥ 180 mg/dl  2hr ≥ 155 mg/dl  3 hr ≥140 mg/dl | 22 weeks | High | None |
| Wahabi (96) | 2013 | Saudi Arabia | Retrospective Cohort | Universal | Fasting blood glucose at booking +/- 100g OGTT at 28-32 weeks | Fasting ≥ 5.3 mmol/l  1 hr ≥ 10.0 mmol/l  2 hr ≥ 8.6 mmol/l  3 hr ≥ 7.8 mmol/l | 24 weeks | High | None |
| Wahabi (97) | 2017 | Saudi Arabia | Prospective Cohort | Universal | Fasting blood glucose ≤ 14 weeks or 75g OGTT at 24-34 weeks | Fasting 5.1-6.9 mmol/l  1 hr ≥ 10.0 mmol/l  2 hr 8.5-11.0 mmol/l | 24 weeks | High | Maternal age, BMI, parity |
| Winsloe (98) | 2025 | United Kingdom | Retrospective cohort | Risk based | Not stated | Not stated | 24 weeks- only term analysis used due to overlap in groups | High | Age: ethnicity, IMD, parity; ethnicity: n/a, IMD: age, ethnicity, parity, pre-existing co-morbidities; BMI: age, ethnicity, IMD, parity, smoking, pre-existing co-morbidities; parity: age, ethnicity, IMD, BMI, pre-existing co-morbidities; smoking: age, ethnicity, IMD, parity, pre-existing co-morbidities; cHTN/pre-existing DM: all variables except cHTN/pre-existing DM, respectively, and pregnancy complications; gHTN/PE/GDM: all variables except gHTN/PE/GDM respectively |
| Xiong (99) | 2001 | Canada | Retrospective cohort | Universal | 50g OGCT at 24-28/40 +/- 100g OGTT | Fasting > 5.8 mmol/L  1 hr > 10.5 mmol/l  2 hr > 9.2 mmol/l  3 hr > 8 mmol/l | Not stated | High | Parity, maternal age, maternal weight, smoking, alcohol use previous neonatal death, previous preterm delivery, previous caesarean section, previous major fetal anomal |
| Young (100) | 2020 | China | Retrospective Cohort | Risk based | 75g OGTT at 24-28 weeks | Fasting ≥ 5.1 mmol/L  1 hr ≥ 10.0 mmol/L  2 hr ≥ 8.5 mmol/L | 24 weeks | Upper Middle | None |
| Zahra (101) | 2022 | Saudi Arabia | Retrospective cohort | Universal | 75g OGTT | FPG 5.1-6.9 mmol/L  2 hr 8.5-11.0 mmol/L | Not stated | High | Not stated |

## Supplementary Table 3: Studies excluded at full text review

| **Author** | **Year** | **Title** | **Reason for exclusion** |
| --- | --- | --- | --- |
| Abdelgadir | 2003 | Factors affecting perinatal morbidity and mortality in pregnancies complicated by diabetes mellitus in Sudan. | Wrong patient population |
| Abell | 2016 | Gestational diabetes mellitus and adverse pregnancy outcomes: The impact of different treatment targets at two major Australian maternity services | Wrong comparator |
| Abell | 2015 | Gestational diabetes mellitus and adverse pregnancy outcomes: Experience from Australia's largest healthcare service | Wrong setting |
| Abella-Del Rosario | 2017 | The prognostic use of uterine artery Doppler velocimetryin predicting adverse pregnancy outcomes among women with gestational diabetes mellitus in a tertiary hospital | Wrong intervention |
| Abell | 1976 | Routine testing for gestational diabetes, pregnancy hypoglycemia and fetal growth retardation, and results of treatment. | Wrong study design |
| Aberg | 1997 | Impaired glucose tolerance during pregnancy is associated with increased fetal mortality in preceding sibs | Wrong study design |
| Abudu | 1987 | Screening for diabetes in pregnancy in a Nigerian population with a high perinatal mortality rate | Wrong outcomes |
| Adams | 1978 | Diabetes in pregnancy: a review | Wrong study design |
| Agbozo | 2018 | Maternal morbidities in Ghana: Risk factors and effect on newborn health outcomes | No full text available |
| Agena | 2019 | Maternal and foetal medical conditions during pregnancy as determinants of intrapartum stillbirth in public health facilities of addis ababa: A case-control study | Wrong outcomes |
| Ajala | 2012 | Diabetes in pregnancy: Mode of delivery and pregnancy outcome: Diabetes in Pregnancy Mother and Baby 3 | No full text available |
| Akhlaghi | 2005 | Comparison of maternal and fetal/neonatal complications in gestational and pre-gestational diabetes mellitus | Wrong comparator |
| Akhter | 1996 | Diabetes in pregnancy in Pakistani women: Prevalence and complications in an indigenous south Asian community | Wrong comparator |
| Al Najashi | 1997 | Congenital anomalies among infants of diabetic mothers: a study of 466 cases at King Fahd Hospital of the University, Al-Khobar. | Wrong outcomes |
| Al Wattar | 2016 | Effect of simple, targeted diet in pregnant women with metabolic risk factors on maternal and fetal outcomes (ESTEEM): study protocol for a pragmatic multicentre randomised trial. | Wrong study design |
| Al Wattar | 2018 | Effects of Mediterranean-style diet in pregnancies with metabolic risk factors (ESTEEM): A pragmatic multicentre randomized trial | Wrong intervention |
| Al-Bassam | 2017 | Universal screening strategy for gestational diabetes mellitus: The experience of Tawam Hospital | Wrong outcomes |
| Al-Dabbous | 1996 | Perinatal morbidity and mortality in offspring of diabetic mothers in Qatif, Saudi Arabia. | Wrong comparator |
| Al-Kadri | 2012 | Factors contributing to intra-uterine fetal death | Wrong study design |
| Alberico | 2017 | Immediate delivery or expectant management in gestational diabetes at term: the GINEXMAL randomised controlled trial. | Wrong outcomes |
| Ali | 2011 | Diabetes in pregnancy: health risks and management. | Wrong study design |
| Allen | 2007 | Teratogenicity associated with pre-existing and gestational diabetes. | Wrong study design |
| Alsulami | 2023 | Complications and risk factors of early-onset versus late-onset gestational diabetes mellitus | Wrong comparator |
| Altman | 1991 | Multicenter survey of diabetic pregnancy in France | Wrong setting |
| Al Khalaf | 2024 | Risk of stillbirth and adverse pregnancy outcomes in a third pregnancy when an earlier pregnancy has ended in stillbirth | Wrong outcomes |
| Al Teheawt | 1995 | Comparative study on: morbidity and mortality among neonates of gestational and frank diabetic mothers. | Wrong study design |
| Ananthan | 2019 | Placental Findings in Singleton Stillbirths: A Case-control Study. | Wrong study design |
| Anonymous | 2003 | Gestational diabetes mellitus | Wrong study design |
| Anonymous | 1992 | Gestational diabetes mellitus | Wrong study design |
| Anonymous | 2022 | Effect of Lifestyle Modification on Pregnancy Outcome for GDM | No full text available |
| Anyaegbunam | 1995 | Chronic hypertension in gestational diabetes: Influence on pregnancy outcome | Wrong intervention |
| Archangelo | 2015 | Identification of clinical comorbidities prevalence in outpatient care high risk pregnancy | Wrong outcomes |
| Arjun | 1998 | Diabetes in pregnancy | Wrong study design |
| Artymuk | 2012 | Stillbirths: Risk factors and main problems of obstetric care | Wrong setting |
| Atkinson | 2022 | Pregnancy-Associated Diabetes Mellitus and Stillbirths by Race and Ethnicity among Hospitalized Pregnant Women in the United States | Wrong outcomes |
| Atarod | 2024 | Evaluation of the Incidence of Gestational Diabetes Mellitus and Some Other Pregnancy Outcomes in Women with Abnormal Fasting Plasma Glucose in the First Trimester of Pregnancy | Wrong comparator |
| Badakhsh | 2016 | Evaluation of maternal and fetal complications in healthy and diabetic pregnant women | Wrong study design |
| Bahaa | 2013 | Comparative study between gestational and pregestational diabetes in relation to glycemic control as regarding fetal and neonatal outcome | Wrong comparator |
| Ball | 2009 | Interim results from the East Anglia Regional Pre-pregnancy care Programme | Wrong patient population |
| Bayraktar | 2001 | Number of relationships between abnormal values in oral glucose tolerance test and adverse pregnancy outcome. | Wrong comparator |
| Barón | 2006 | Prenatal risk factors in late fetal death | Wrong outcomes |
| Basha | 2019 | Prevalence of gestational diabetes and contributing factors among pregnant Jordanian women attending Jordan University Hospital. | Wrong outcomes |
| Battarbee | 2020 | The association of pregestational and gestational diabetes with severe neonatal morbidity and mortality | Wrong outcomes |
| Bawah | 2019 | Gestational diabetes mellitus and obstetric outcomes in a Ghanaian community | Wrong study design |
| Becerra | 1990 | Diabetes mellitus during pregnancy and the risks for specific birth defects: a population-based case-control study. | Wrong patient population |
| Beinder | 2008 | Stillbirth and sudden infant death syndrome. Common factors and differences | Wrong study design |
| Beischer | 1996 | Identification and treatment of women with hyperglycaemia diagnosed during pregnancy can significantly reduce perinatal mortality rates. | Wrong outcomes |
| Bell | 2011 | Congenital anomalies in offspring of mothers with pre-gestational diabetes | Wrong patient population |
| Bell | 2011 | Risk of recurrent adverse pregnancy outcome in women with diabetes | Wrong patient population |
| Bell | 2010 | Outcome of pregnancies complicated by gestational diabetes: A multi-centre study from the North East of England | Wrong study design |
| Bell | 2010 | Diagnosis, treatment and outcome of gestational diabetes: A multi-centre study in north-east England (NorGES) | Wrong study design |
| Berger | 2016 | Diabetes in Pregnancy. | Wrong study design |
| Berggren | 2012 | Perinatal outcomes in Hispanic and non-Hispanic white women with mild gestational diabetes. | Wrong comparator |
| Berggren | 2012 | The NICHD-MFMU GDM study: Differences in perinatal outcomes between Hispanic women and Non-Hispanic White women with and without gestational diabetes | Wrong comparator |
| Beyerlein | 2010 | Improvement in pregnancy-related outcomes in the offspring of diabetic mothers in Bavaria, Germany, during 1987-2007 A. Beyerlein et al. Pregnancy-related outcomes in offspring of diabetic mothers. | Wrong outcomes |
| Bezhenar | 2020 | Stillbirth: Prerequisites and risk factors | Wrong outcomes |
| Bezhenar | 2021 | Risk factors for perinatal loss - Reality or fiction? | Wrong study design |
| Bhat | 2012 | Outcome of gestational diabetes mellitus from a tertiary referral center in South India: a case-control study | Wrong outcomes |
| Bhorat | 2018 | Determination of the fetal myocardial performance index in women with gestational impaired glucose tolerance and to assess whether this parameter is a possible prognostic indicator of adverse fetal outcome. | Wrong intervention |
| Bhowmik | 2016 | First Trimester complications in pregnancy with diabetes. | Wrong study design |
| Billionnet | 2017 | Gestational diabetes and adverse perinatal outcomes from 716,152 births in France in 2012 | Wrong study design |
| Blachier | 2014 | Diet or medically treated gestational diabetes: Is there any difference for obstetrical and neonatal complications? A French cohort study | Wrong setting |
| Black | 2013 | Maternal Obesity and the Risk of Stillbirth | Wrong study design |
| Bogdanet | 2017 | ATLANTIC DIP: insulin therapy for women with IADPSG-diagnosed gestational diabetes mellitus. Does it work? | Wrong comparator |
| Boriboonhirunsarn | 2006 | Adverse pregnancy outcomes in gestational diabetes mellitus | Wrong outcomes |
| Bordin | 2020 | Gestational diabetes mellitus yesterday, today and tomorrow: A 13-year Italian cohort study. | Wrong comparator |
| Brackett | 2022 | Factors Associated with Occurrence of Stillbirth before 32 Weeks of Gestation in a Contemporary Cohort | Wrong study design |
| Brailovschi | 2012 | Risk factors for intrapartum fetal death and trends over the years | Wrong study design |
| Brehm Christensen | 2016 | Higher rate of serious perinatal events in non-Western women in Denmark. | Wrong comparator |
| Brokaw | 2018 | Does a History of Gestational Diabetes Mellitus Affect Key Outcomes in Women Participating in a Diabetes Prevention Program? | Wrong outcomes |
| Brown | 2017 | Exercise for pregnant women with gestational diabetes for improving maternal and fetal outcomes. | Wrong study design |
| Bukowski | 2014 | Fetal Growth and Risk of Stillbirth: A Population-Based Case-Control Study | Wrong outcomes |
| Bunak | 2016 | Pregnancy outcomes in women with hyperglycemia in the morning | Wrong intervention |
| Cahill | 2015 | Markers of Glycemic Control and Neonatal Morbidity in High-Risk Insulin-Resistant Pregnancies | Wrong outcomes |
| Caliskan | 2019 | Determination of the Effect of Obstetric Variables on Stillbirths: A Case Control Study in Turkey | Wrong study design |
| Campbell | 2009 | Placental dysfunction and stillbirth in gestational diabetes mellitus | Wrong setting |
| Capula | 2013 | HbA1c levels in patients with gestational diabetes mellitus: Relationship with pre-pregnancy BMI and pregnancy outcome. | Wrong comparator |
| Cassimatis | 2018 | Causes and timing of stillbirth among women with pre-gestational and gestational diabetes: Stillbirth Collaborative Research Network data | Wrong setting |
| Cauldwell | 2023 | Screening for Late-Onset Gestational Diabetes: Are There Any Clinical Benefits | Wrong outcomes |
| Ceysens | 2016 | Exercise for pregnant women with gestational diabetes for improving maternal and fetal outcomes | Wrong study design |
| Challis | 2002 | Gestational diabetes mellitus and fetal death in Mozambique: an incident case‐referent study | Wrong study design |
| Chehab | 2023 | Glycemic Control Trajectories and Risk of Perinatal Complications Among Individuals With Gestational Diabetes | Wrong outcomes |
| Chen | 2019 | Diabetes in pregnancy in associations with perinatal and postneonatal mortality in First Nations and non-Indigenous populations in Quebec, Canada: population-based linked birth cohort study. | Wrong outcomes |
| Chen | 2001 | Effect of gestational impaired glucose tolerance on pregnant outcome | Wrong study design |
| Chiu | 2019 | Pregnancy outcomes of women with previous gestational diabetes attending a dedicated virtual service in a London maternity unit | Wrong patient population |
| Cho | 2022 | Adverse Pregnancy Outcomes and Maternal Chronic Diseases in the Future: A Cross-Sectional Study Using KoGES-HEXA Data. | Wrong outcomes |
| Choi | 2021 | The Korean Pregnancy Outcome Study (KPOS): Study Design and Participants. | Wrong study design |
| Clarke | 2020 | Early Pregnancy Screening for Women at High-Risk of GDM Results in Reduced Neonatal Morbidity and Similar Maternal Outcomes to Routine Screening. | Wrong intervention |
| Corcoran | 2011 | Neonatal outcome in pregnancies complicated by gestational diabetes | Wrong comparator |
| Coustan | 2015 | Gestational diabetes mellitus | Wrong study design |
| Coustan | 2013 | Gestational diabetes mellitus | Wrong study design |
| Cozzolino | 2019 | Ongoing pregnancies in patients with unexplained recurrent pregnancy loss: adverse obstetric outcomes. | Wrong outcomes |
| Crippa | 2014 | Outcome of induction of labour in women with gestational diabetes and comparison with general obstetric population | Wrong outcomes |
| Cundy | 2006 | Why do poor outcomes persist in diabetic pregnancy? | Wrong patient population |
| Dafallah | 2004 | Diabetes mellitus during pregnancy. Fetal outcome | Wrong patient population |
| Dafallah | 2004 | Risk factors predisposing to abruptio placentae. Maternal and fetal outcome. | Wrong outcomes |
| Dagdeviren | 2022 | Application of the international classification of diseases-perinatal mortality (ICD-PM) system to stillbirths: A single center experience in a middle income country | Wrong study design |
| Dah | 2023 | Stillbirth incidence and determinants in a tertiary health facility in the Volta Region of Ghana | Wrong outcomes |
| Dahlstrom | 2021 | Maternal Diabetes and Obesity | Wrong study design |
| Darling | 2014 | Maternal hyperglycemia and adverse pregnancy outcomes in Dar es Salaam, Tanzania | Wrong comparator |
| Davis | 2013 | A threefold increase in gestational diabetes over two years: Review of screening practices and pregnancy outcomes in Indigenous women of Cape York, Australia | Wrong outcomes |
| De Almeida | 2007 | Risk-factors for antepartum fetal deaths in the city of São Paulo, Brazil | Wrong outcomes |
| De Graaf | 2017 | Risk factors for stillbirth in a socio-economically disadvantaged urban Australian population. | Wrong study design |
| DeVeciana | 2011 | How does the degree of hyperglycemia recorded during glucose tolerance testing for gestational diabetes impact perinatal outcome? | Wrong setting |
| Di Mario | 2007 | Risk factors for stillbirth in developing countries: A systematic review of the literature | Wrong study design |
| Djagadou | 2019 | [Diagnostic, therapeutic, and prognostic features of gestational diabetes at the Sylvanus Olympio University Hospital Center]. | Wrong study design |
| Djomhou | 2016 | Maternal hyperglycemia during labor and related immediate post-partum maternal and perinatal outcomes at the Yaounde Central Hospital, Cameroon. | Wrong study design |
| Dodd | 2017 | Risk assessment and management of diabetes in pregnancy and associated stillbirth - An audit in North Bristol | Wrong setting |
| Dornhorst | 2008 | Will the NICE guidelines on diabetes in pregnancy improve outcomes? | Wrong study design |
| Driul | 2010 | Neonatal and maternal outcomes by gestational diabetes mellitus and impaired glucose tolerance: A retrospective analysis of our 6-years experience | Wrong setting |
| Duarte | 2009 | Gestational diabetes - Influence of gestational age of diagnosis and family history of diabetes | Wrong patient population |
| Dudley | 2007 | Diabetic-Associated Stillbirth: Incidence, Pathophysiology, and Prevention | Wrong study design |
| Dunne | 2000 | Fetal and maternal outcomes in Indo-Asian compared to Caucasian women with diabetes in pregnancy. | Wrong comparator |
| Duong | 2015 | Pregnancy and neonatal outcomes in Indigenous Australians with diabetes in pregnancy | Wrong study design |
| Dyck | 2020 | Epidemiology of Diabetes in Pregnancy Among First Nations and Non-First Nations Women in Saskatchewan, 1980-2013. Part 2: Predictors and Early Complications; Results From the DIP: ORRIIGENSS Project. | Wrong outcomes |
| Edwards | 2013 | Differences in post-mortem findings after stillbirth in women with and without diabetes. | Wrong patient population |
| Egan | 2020 | A core outcome set for studies of gestational diabetes mellitus prevention and treatment. | Wrong study design |
| Ekekwe | 2012 | Perinatal mortality and associated risk factors at Lagos University Teaching Hospital | Wrong outcomes |
| Ekwueme | 2018 | Hyperglycemia and beta cell function in pregnancy | Data unavailable |
| Esakoff | 2011 | Perinatal outcomes in patients with gestational diabetes mellitus by race/ethnicity. | Wrong setting |
| Esakoff | 2017 | Does small for gestational age worsen outcomes in gestational diabetics?. | Wrong comparator |
| Essadi | 2010 | Outcomes of pregnancies complicated by gestational diabetes mellitus in Misurata | Wrong outcomes |
| Fadl | 2012 | Outcomes of gestational diabetes in Sweden depending on country of birth | Wrong outcomes |
| Facchinetti | 2011 | A multicenter, case-control study on risk factors for antepartum stillbirth | Wrong outcomes |
| Faiz | 2012 | Trends and risk factors of stillbirth in New Jersey 19972005 | Wrong outcomes |
| Farrar | 2019 | Re: Gestational diabetes and the risk of late stillbirth: a case-control study from England, UK. | Duplicate |
| Feleke | 2017 | Determinants of gestational diabetes mellitus: a case-control study | Wrong setting |
| Feleke | 2021 | Progression of pregnancy induced diabetes mellitus to type two diabetes mellitus, an ambidirectional cohort study. | Wrong outcomes |
| Figueroa | 2013 | Relationship between 1-hour glucose challenge test results and perinatal outcomes. | Wrong outcomes |
| Flenady | 2021 | Making every birth count: Outcomes of a perinatal mortality audit program. | Wrong study design |
| Forsbach | 1999 | [Maternal and fetal morbidity in a group of women with gestational diabetes]. | Wrong outcomes |
| Fraser | 2006 | Gestational diabetes: After the ACHOIS trial | Wrong study design |
| Fretts | 2010 | Stillbirth epidemiology, risk factors, and opportunities for stillbirth prevention | Wrong study design |
| Fretts | 1997 | Causes of fetal death in women of advanced maternal age | Wrong outcomes |
| Friedman | 2016 | Stillbirth and neonatal adverse outcomes in pregnancies complicated by preexisting and gestational diabetes | Wrong patient population |
| Friedman | 2016 | Timing of delivery for gestational diabetes mellitus | Wrong intervention |
| Gabbe | 1993 | Pregnancy in women with diabetes mellitus | Wrong study design |
| Gabbe | 2007 | Diabetes mellitus in pregnancy | Wrong study design |
| Gabbe | 1977 | Management and outcome of pregnancy in diabetes mellitus, Classes B to R | Wrong study design |
| Gai | 1990 | Intrauterine deaths: a clinical and pathologic analysis | Wrong study design |
| Ganchev | 1982 | Diabetes mellitus in pregnancy and perinatal infant mortality | Full text unavailable |
| Garcia | 2021 | Analysis of routinely collected data: Determining associations of maternal risk factors and infant outcomes with gestational diabetes, in Pakistani, Indian, Bangladeshi and white British pregnant women in Luton, England | Wrong outcomes |
| Gardosi | 2013 | Maternal and fetal risk factors for stillbirth: Population based study | Wrong setting |
| Genc | 2014 | Evaluation of obstetric outcomes and risk factors of  early late and term stillbirths. | Wrong study design |
| Geisler | 2013 | Pregnancy after previous stillbirth | Wrong patient population |
| Geng | 2024 | Association of Parity with the Risks of Gestational Diabetes and Macrosomia: A Retrospective Cohort Study in Nanjing, China | Wrong outcomes |
| George | 2015 | Comparison of neonatal outcomes in women with gestational diabetes with moderate hyperglycaemia on metformin or glibenclamide--a randomised controlled trial. | Wrong setting |
| Ghosh | 2013 | Maternal and neonatal outcomes in gestational diabetes mellitus | Wrong outcomes |
| Girz | 1992 | Sudden fetal death in women with well-controlled, intensively monitored gestational diabetes. | Wrong setting |
| Godel | 1975 | Diabetes in pregnancy. A clinical and statistical analysis of 1800 pregnancies and deliveries from 1952-71. II. Perinatal mortality in relation to the treatment of diabetes and the frequency of toxemia and urinary tract infections | No full text available |
| Gokaslan | 2003 | Pregnancy and diabetes | Wrong study design |
| Gonzalez-Quintero | 2007 | The impact of glycemic control on neonatal outcome in singleton pregnancies complicated by gestational diabetes. | Wrong comparator |
| Greco | 2024 | Gestational diabetes mellitus and adverse maternal and perinatal outcomes in twin and singleton pregnancies: a systematic review and meta-analysis | Wrong study design |
| Guerrero Cervera | 2019 | Alterations of carbohydrate metabolism during pregnancy. Effects on the mother and new born infant | No full text available |
| Gui | 2014 | Association between hyperglycemia in middle and late pregnancy and maternal-fetal outcomes: a retrospective study. | Wrong setting |
| Gunnarsson | 2012 | Gestational diabetes in Iceland 2007.2008 | Wrong outcomes |
| Guosheng | 2009 | The relationship of serum AGE levels in diabetic mothers with adverse fetal outcome. | Data unavailable |
| Gupta | 2018 | A cross sectional observational study to assess the causes of stillbirth in developing country | Wrong study design |
| Gurol-Urganci | 2022 | Obstetric interventions and pregnancy outcomes during the COVID-19 pandemic in England: A nationwide cohort study. | Wrong outcomes |
| Gwako | 2021 | Association between obstetric and medical risk factors and stillbirths in a low-income urban setting. | Wrong study design |
| Hadden | 2010 | Clinical problems in diabetic pregnancy | Wrong study design |
| Haliloğlu | 2020 | The effect of treatment on outcomes in gestational diabetes mellitus | Wrong intervention |
| Halla | 2015 | Diabetes mellitus in pregnancy, still changing | Wrong study design |
| Hanson | 2022 | Screening of Gestational Diabetes and Its Risk Factors: Pregnancy Outcome of Women with Gestational Diabetes Risk Factors According to Glycose Tolerance Test Results | Wrong outcomes |
| Hawthorne | 1994 | Outcome of diabetic pregnancy and glucose intolerance in pregnancy: an audit of fetal loss in Newcastle General Hospital 1977-1990. | Wrong outcomes |
| Helgadottir | 2011 | Incidence and risk factors of fetal death in Norway: a case-control study | Wrong study design |
| Hillier | 2021 | A Pragmatic, Randomized Clinical Trial of Gestational Diabetes Screening. | Wrong outcomes |
| Hirst | 2012 | Consequences of gestational diabetes in an urban hospital in Viet Nam: A prospective cohort study | Wrong outcomes |
| Hochberg | 2019 | Perinatal outcome following induction of labor in patients with good glycemic controlled gestational diabetes: does timing matter? | Wrong setting |
| Hod | 2017 | The relevance of hyperglycemia in pregnancy on perinatal outcomes and future burden of non-communicable diseases in Europe and the Figo guideline | Wrong study design |
| Holst | 2016 | Fertility problems and risk of gestational diabetes mellitus: a nationwide cohort study. | Wrong comparator |
| Hummel | 2023 | Diabetes and pregnancy | Wrong study design |
| Ijas | 2019 | Independent and concomitant associations of gestational diabetes and maternal obesity to perinatal outcome: A register-based study | Wrong outcomes |
| Immanuel | 2021 | Pregnancy outcomes among multi-ethnic women with different degrees of hyperglycaemia during pregnancy in an urban New Zealand population and their association with postnatal HbA1c uptake. | Wrong outcomes |
| Jagannatham | 2024 | An Electronic Medical Record Intervention to Increase Early Screening Rates for Gestational Diabetes | Wrong outcomes |
| Jahani | 2015 | Factors affecting stillbirth rate in the hospitals affiliated to Babol University of Medical Sciences | Wrong study design |
| Jeyaparam | 2024 | Retrospective cohort study of the association between socioeconomic deprivation and incidence of gestational diabetes and perinatal outcomes | Wrong outcomes |
| Jovanovic | 2005 | Elevated pregnancy losses at high and low extremes of maternal glucose in early normal and diabetic pregnancy: Evidence for a protective adaptation in diabetes | Wrong patient population |
| Jovic | 2016 | Characteristics of pregnancy, delivery and the postpartum period in pregnant women diagnosed with gestational diabetes mellitus | Wrong outcomes |
| Kalyan | 2018 | Prevalence of diabetic foetopathy in patients with gestational diabetes in rural set up in India | Wrong study design |
| Kampan | 2013 | Outcome of pregnancy among Malaysian women with diabetes mellitus - A single centre experience | Wrong patient population |
| Kapustin | 2020 | Time and mode of delivery in diabetic pregnancy: a review. | Wrong study design |
| Karalasingam | 2014 | Lethal congenital malformation in pre-existing diabetes and gestational diabetes mellitus | Wrong setting |
| Kavari | 2009 | The study of factors related to pregnancy diabetes in women with pregnancy diabetes referred to Shiraz Diabetic Centre | Wrong patient population |
| Kazmi | 2012 | Maternal and neonatal outcomes of gestational diabetes mellitus | Full text unavailable |
| Keller-Wood | 2014 | Elevated maternal cortisol leads to relative maternal hyperglycemia and increased stillbirth in ovine pregnancy. | Wrong patient population |
| Kerby | 2021 | Placental morphology and cellular characteristics in stillbirths in women with diabetes and unexplained stillbirths | Wrong outcomes |
| Khatun | 2005 | Infant outcomes of gestational diabetes mellitus | Wrong study design |
| Kiefer | 2022 | Gestational Weight Gain and Adverse Maternal and Neonatal Outcomes for Pregnancies Complicated by Pregestational and Gestational Diabetes | Wrong comparator |
| Kilcullen | 2022 | Stillbirth risks and rates for Aboriginal and Torres Strait Islander women and their babies in North Queensland | Wrong outcomes |
| Kim | 2018 | The trends and risk factors to predict adverse outcomes in gestational diabetes mellitus: A 10-year experience from 2006 to 2015 in a single tertiary center | Wrong comparator |
| Kleiner | 2020 | Pregnancy outcomes in association with placental histopathology in pregnancies complicated by macrosomia in diabetic vs. non-diabetic women | Wrong outcomes |
| Kodama | 2014 | Placental pathology of stillbirths complicated by glucose intolerance during pregnancy: A regional population-based study in Japan | Wrong patient population |
| Kodama | 2013 | Impact of new gestational diabetes mellitus criteria on stillbirth: a regional population-based study in Japan. | Wrong intervention |
| Kosman | 2016 | Perinatal outcomes in gestational diabetes in relation to ethnicity in the Netherlands | Wrong outcomes |
| Kragelund Nielsen | 2021 | Migration, Gestational Diabetes, and Adverse Pregnancy Outcomes: A Nationwide Study of Singleton Deliveries in Denmark | Wrong outcomes |
| Kunjachen Maducolil | 2018 | Risk factors and classification of stillbirth in a Middle Eastern population: a retrospective study. | Wrong study design |
| Landon | 2007 | The National Institute of Child Health and Human Development Maternal-Fetal Medicine Unit Network randomized clinical trial in progress: Standard therapy versus no therapy for mild gestational diabetes | Wrong study design |
| Landon | 2009 | A multicenter, randomized trial of treatment for mild gestational diabetes. | Wrong setting |
| Langer | 1994 | Intensified versus conventional management of gestational diabetes. | Wrong outcomes |
| Langer | 2004 | Gestational diabetes: the consequences of not treating | Wrong study design |
| Lankala | 2023 | Perinatal outcomes of pregnancy with gestational diabetes in a secondary care hospital in Tamil Nadu, India | Wrong comparator |
| Lapolla | 2009 | Gestational diabetes mellitus in Italy: a multicenter study. | Wrong setting |
| Lappharat | 2022 | A model for predicting gestational diabetes mellitus in early pregnancy: a prospective study in Thailand. | Wrong outcomes |
| Lau | 1994 | A perinatal audit of stillbirths in a teaching hospital in Hong Kong. | Wrong study design |
| Lee | 2015 | Outcomes of late preterm pregnancies complicated by gestational diabetes mellitus and polyhydramnios | Wrong patient population |
| Lemieux | 2022 | The association between gestational diabetes and stillbirth: a systematic review and meta-analysis. | Wrong study design |
| Leon | 2015 | Rate of Gestational Diabetes Mellitus and Pregnancy Outcomes in Patients with Chronic Hypertension | Wrong outcomes |
| Leung | 2018 | Adverse pregnancy outcomes with glyburide vs insulin among patients with gestational diabetes established by the International Association of Diabetes and Pregnancy Study Group (IADPSG) | Wrong outcomes |
| Liang | 2024 | Outcome of Pregnancy Oral Glucose Tolerance Test and Preterm Birth | Wrong outcomes |
| Little | 2021 | Changes in Delivery Timing for High-Risk Pregnancies in the United States. | Wrong outcomes |
| Liu | 2023 | The adverse effect of gestational diabetes mellitus and hypertensive disorders of pregnancy on maternal–perinatal outcomes among singleton and twin pregnancies: a retrospective cohort study (2011–2019) | Wrong outcomes |
| Liu | 2013 | Analysis of intrauterine fetal demise-A hospital-based study in Taiwan over a decade | Wrong outcomes |
| Liu | 2022 | Determinants for Perinatal Mortality in South China: A Prospective Cohort Study | Wrong outcomes |
| Li | 2022 | Effects of gestational hyperglycemia on maternal and infant health and its management strategy | Wrong outcomes |
| Li | 2021 | Independent and cumulative effects of risk factors associated with stillbirths in 50 low- and middle-income countries: A multi-country cross-sectional study | Wrong outcomes |
| Lorenz | 2023 | Risk of stillbirth in maternal (gestational) diabetes mellitus | Wrong outcomes |
| Lorenz | 2023 | Risk of stillbirth in pregnancies complicated by maternal diabetes or gestational diabetes | Wrong study design |
| Loukovaara | 2007 | Fetal risks of maternal diabetes | Wrong study design |
| Lynch | 2022 | Stillbirth in women with diabetes: a retrospective analysis of fetal autopsy reports | Wrong study design |
| László | 2013 | Maternal bereavement during pregnancy and the risk of stillbirth: A nationwide cohort study in Sweden: Editorial comment | Wrong study design |
| Ma | 2023 | Effect of birth interval on maternal and infant outcomes in patients with gestational diabetes mellitus | Wrong study design |
| Mackenzie | 2021 | Pregnancy and development of diabetes in First Nations and non-First Nations women in Alberta, Canada | Wrong study design |
| Maheswara | 2023 | Maternal and Perinatal Outcomes in Pregnancy Complicated with Pre- and Gestational Diabetes Mellitus | Wrong outcomes |
| Makwana | 2021 | Prevalence and causes of stillbirths at a tertiary care hospital: One year study | Wrong outcomes |
| Maleki | 2021 | Incidence and maternal-fetal risk factors of stillbirth. A population-based historical cohort and a nested casecontrol study | Wrong study design |
| Mallins | 1978 | Congenital malformations and fetal mortality in diabetic pregnancy | Wrong patient population |
| Man | 2016 | Contemporary demographic features of intrauterine death: A review of >1,000 stillbirths and intrauterine deaths in London | Wrong comparator |
| Mannan | 2012 | Prevalence and pregnancy outcome of gestational diabetes mellitus among Bangladeshi urban pregnant women | Wrong outcomes |
| Mantovani | 2017 | Early diagnosis, active management and early induction of labour in pre-existing and gestational diabetes: Effects on pregnancy and delivery outcomes | Wrong patient population |
| Mao | 2023 | Analysis of risk factors related to 652 stillbirths and evaluation of prevention and control measures | No full text available |
| Marathe | 2014 | Gestational diabetes and obstetric cholestasis: A 10 year retrospective cohort review of outcomes and predictors | Wrong comparator |
| Mathieu | 2023 |  | Wrong study design |
| Mathews | 2011 | Retrospective cohort study comparing neonatal outcomes of women treated with glyburide or insulin in gestational diabetes: A 5-year experience in a South Indian teaching hospital | Wrong outcomes |
| McElwee | 2023 | Risk of Stillbirth in Pregnancies Complicated by Diabetes, Stratified by Fetal Growth | Wrong study design |
| McLaughlin | 2009 | Associations among health literacy levels and health outcomes in pregnant women with pregestational and gestational diabetes in an urban setting. | Wrong outcomes |
| McLean | 2014 | Metformin treatment of gestational diabetes results in similar glycemic control and infant outcomes but less maternal weight gain than insulin treatment | Wrong comparator |
| Mclennan | 2024 | Impact of COVID-19 on gestational diabetes pregnancy outcomes in the UK: A multicentre retrospective cohort study | Wrong study design |
| McMahon | 1998 | Gestational diabetes mellitus. Risk factors, obstetric complications and infant outcomes. | Wrong outcomes |
| Meek | 2015 | Diagnosis of gestational diabetes mellitus: falling through the net | Wrong outcomes |
| Melchor | 2019 | Effect of maternal obesity on pregnancy outcomes in women delivering singleton babies: a historical cohort study. | Wrong outcomes |
| Mesdaghinia | 2013 | Comparison of newborn outcomes in women with gestational diabetes mellitus treated with metformin or insulin: A randomised blinded trial | Wrong comparator |
| Mesleh | 1986 | Stillbirths and first week deaths in Saudi Arabia: Causes and prevention | Wrong outcomes |
| Metcalfe | 2017 | Trends in obstetric intervention and pregnancy outcomes of Canadian women with diabetes in pregnancy from 2004 to 2015 | Wrong outcomes |
| Michelsson | 1968 | Perinatal mortality in diabetic pregnancy | Wrong patient population |
| Milani | 2023 | Assessment of neonatal and maternal complications in pregnant women with gestational diabetes in the Iranian population | Wrong intervention |
| Mills | 2015 | Comparison of pregnancy outcomes between non-treated women with gestational diabetes (GDM) diagnosed by International Association of Diabetes and Pregnancy Study Groups (IADPSG) criteria compared to women without gestational diabetes | Wrong comparator |
| Mirghani | 2000 | A simplified management of diabetic pregnant women | Wrong study design |
| Mirzamoradi | 2015 | Investigating the effects of treatment based on single high blood glucose in gestational diabetes screening on maternal and neonatal complications. | Wrong study design |
| Mistry | 2021 | Gestational diabetes mellitus (GDM) and adverse pregnancy outcome in South Asia: A systematic review. | Wrong study design |
| Miu | 2014 | What is the optimal gestational age for women with gestational diabetes type A1 to deliver?. | Wrong outcomes |
| Mogibian | 2010 | Incidence of gestational diabetes mellitus in pregnant women | Wrong outcomes |
| Mohamed Ismail | 2013 | Homeostatic indices of insulin resistance among gestational diabetics in anticipating pregnancy complications | Wrong outcomes |
| Mohamed | 2015 | Pattern of glucose intolerance among pregnant women with unexplained IUFD | Wrong outcomes |
| Mohsin | 2006 | The influence of antenatal and maternal factors on stillbirths and neonatal deaths in New South Wales, Australia | Wrong outcomes |
| Monteiro | 2022 | Maternofetal outcomes in early-onset gestational diabetes: does weight gain matter? | Wrong outcomes |
| Moore | 2007 | Metformin and insulin in the management of gestational diabetes mellitus: Preliminary results of a comparison | Wrong outcomes |
| Moorthy | 2017 | An analysis of pregnancy and neonatal outcomes in non-treated and treated women with gestational diabetes (GDM) diagnosed by the International Association of the Diabetes Pregnancy Study Groups (IADPSG) criteria and women without gestational diabetes | Wrong intervention |
| Mounika | 2015 | Study of 100 cases of gestational diabetes in Sree Balaji Medical College and Hospital (SBMCH), Tamil Nadu, India | Wrong comparator |
| Moura | 2014 | Risk factors for perinatal death in two different levels of care: A case-control study | Wrong outcomes |
| Muin | 2022 | Universal Gestational Diabetes Screening and Antepartum Stillbirth Rates in Austria - A Population-Based Study | Wrong study design |
| Myskowski | 2022 | Influence of gestational diabetes in twin pregnancy on the condition of newborns and early neonatal complications. | Data unavailable |
| Nabi | 2022 | Effect of Overt Diabetes and Gestational Diabetes Mellitus on Pregnancy Outcomes and Progression | Wrong comparator |
| Nakabuye | 2017 | Prevalence of hyperglycaemia first detected during pregnancy and subsequent obstetric outcomes at St. Francis Hospital Nsambya. | Wrong patient population |
| Nandini | 2015 | Gestational diabetes mellitus - Its control, maternal and perinatal outcome - A retrospective study in Sri Balaji Medical College Hospital, Chennai, Tamil Nadu, India | Wrong outcomes |
| Nashif | 2023 | Neonatal outcomes and rationale for timing of birth in perinatal diabetes: a retrospective cohort study | Wrong outcomes |
| Natamba | 2019 | Burden, risk factors and maternal and offspring outcomes of gestational diabetes mellitus (GDM) in sub-Saharan Africa (SSA): a systematic review and meta-analysis. | Wrong study design |
| Newtonraj | 2017 | Level, causes, and risk factors of stillbirth: A population-based case control study from Chandigarh, India | Wrong outcomes |
| Ngai | 2014 | Outcome of pregnancy when gestational diabetes mellitus is diagnosed before or after 24 weeks of gestation | Wrong outcomes |
| Nie | 2016 | A retrospective study of maternal and neonatal outcomes in overweight and obese women with gestational diabetes mellitus | Wrong patient population |
| Niemi | 2013 | Adverse perinatal outcomes stratified by birthweight in pregnancies with and without gestational diabetes | Wrong setting |
| Niu | 2014 | What is the optimal gestational age for women with gestational diabetes type A1 to deliver? | Wrong outcomes |
| Nobumoto | 2015 | Effect of the new diagnostic criteria for gestational diabetes mellitus among Japanese women | Wrong comparator |
| O'Sullivan | 1973 | Gestational diabetes and perinatal mortality rate. | Wrong outcomes |
| Obadi | 2018 | Risk factors of stillbirth in Yemen | Wrong study design |
| Odibo | 2009 | Maternal and pregnancy variables affecting stillbirth | Wrong patient population |
| Oniya | 2011 | Outcomes of pregnancies complicated by diabetes | Wrong patient population |
| Onuoha | 2024 | The simultaneous occurrence of gestational diabetes and hypertensive disorders of pregnancy affects fetal growth and neonatal morbidity | Wrong study design |
| Oo | 2013 | The management and the outcome of gestational diabetic women in North tees and Hartlepool NHS trust, United Kingdom: Departmental Audit 2012 | Wrong study design |
| Oster | 2015 | A Retrospective Analysis of Stillbirth Epidemiology and Risk Factors Among First Nations and Non-First Nations Pregnancies in Alberta From 2000 to 2009 | Wrong comparator |
| Osuagwu | 2020 | Adverse Maternal Outcomes of Fijian Women with Gestational Diabetes Mellitus and the Associated Risk Factors | Wrong outcomes |
| Özekinci | 2011 | Abnormal One-Hour 5 -Gram Glucose Challenge Test and Perinatal Outcomes. | Wrong outcomes |
| Packer | 2022 | Increased Rates of Adverse Perinatal Outcomes in Women With Gestational Diabetes and Depression | Wrong study design |
| Page | 2020 | Characteristics of Stillbirths Associated With Diabetes in a Diverse U.S. Cohort. | Wrong outcomes |
| Panpitpat | 2015 | The effects of systematic management on maternal and neonatal complications in gestational diabetes subjects | Wrong outcomes |
| Papola | 2024 | Risk Factors for Stillbirth: Time to Stop, Rethink, Act, and Let Every Baby Count | Wrong study design |
| Parrentini | 2020 | Gestational diabetes: A link between OGTT, maternal-fetal outcomes and maternal glucose tolerance after childbirth | Wrong comparator |
| Patel | 2018 | Outcome in gestational diabetes mellitus after various treatment modality: A tertiary center experience in North India | Duplicate |
| Pendek | 2010 | GDM: Risk factors (RF) and pregnancy outcomes (PO) of high risk patients attending university of Malaya medical centre (UMMC) antenatal clinic (ANC) service | Wrong setting |
| Piffer | 2017 | The prevalence of gestational diabetes in the province of Trento, trend 2010-2015 and adverse neonatal outcomes | No full text available |
| Ples | 2010 | Pregnancy hyperglycemia - Predictive factor for perinatal complications | No full text available |
| Posner | 1971 | The outcome of pregnancy in class A diabetes mellitus | Wrong outcomes |
| Preda | 2021 | Analysis of maternal and neonatal complications in a group of patients with gestational diabetes mellitus | Wrong outcomes |
| Prüst | 2023 | Risk factor-based screening compared to universal screening for gestational diabetes mellitus in marginalized Burman and Karen populations on the Thailand-Myanmar border: An observational cohort | Wrong outcomes |
| Qadir | 2012 | Maternal and foetal outcome in gestational diabetes. | Wrong comparator |
| Ramji | 2024 | The impact of isolated obesity compared with obesity and other risk factors on risk of stillbirth: a retrospective cohort study | Wrong outcomes |
| Ramsay | 2017 | Applying ACHOIS in the northern hemisphere: A 3-year audit of maternal and fetal outcomes in women with gestational diabetes (GDM) in western Scotland | Wrong comparator |
| Randrianaivo | 2006 | Study of 178 ante partum deaths in 2001-2004 in the southern part of Reunion Island | Wrong study design |
| Reed | 2015 | Maternal hyperglycemia exacerbates vasoconstriction in fetal-placental arteries in an ex-vivo placenta perfusion model: A possible mechanism for perinatal mortality in pregnancy complicated by diabetes mellitus | Wrong study design |
| Richey | 1995 | Observations concerning 'unexplained' fetal demise in pregnancy complicated by diabetes mellitus | Wrong study design |
| Roopnarinesingh | 1976 | Foetal loss in pregnant diabetic women | Wrong outcomes |
| Rosenn | 1998 | Antenatal fetal testing in pregnancies complicated by Gestational Diabetes Mellitus: Why, who and how? | Wrong outcomes |
| Rossi | 2019 | 725: Identifiable risk factors associated with early stillbirth | Wrong study design |
| Rossouw | 2017 | An audit of stillborn babies in mothers with diabetes mellitus at a tertiary South African Hospital | Wrong patient population |
| Rowan | 2008 | Metformin versus insulin for the treatment of gestational diabetes | Wrong comparator |
| Rudge | 2000 | Perinatal outcome of pregnancies complicated by diabetes and by maternal daily hyperglycemia not related to diabetes: A retrospective 10-year analysis | Wrong patient population |
| Rudland | 2012 | Gestational Diabetes: Seeing Both the Forest and the Trees | Wrong study design |
| Rust | 2015 | Evaluation of risk factors for and effects of gestational diabetes mellitus on mothers and newborn in Austria | Wrong outcomes |
| Sacco | 2015 | Can we manage diet controlled GDM in the community? Outcomes and experience from the Royal London Hospital 2011-2013 | Wrong comparator |
| Salamon | 2013 | [Comparison of the effectiveness of two internationally recommended screening methods for the diagnosis of gestational diabetes]. | Wrong outcomes |
| Saleem | 2014 | A prospective study of maternal, fetal and neonatal deaths in low- and middle-income countries | Wrong study design |
| Sargent | 2018 | The cost-effectiveness of various antenatal surveillance strategies in preventing intrauterine fetal demise in pregnancies complicated by gestational diabetes A2 | Wrong intervention |
| Satyajit | 2017 | Study of prevalence, maternal and perinatal outcome in gestational diabetes in rural population | Wrong outcomes |
| Sauvegrain | 2020 | Understanding high rates of stillbirth and neonatal death in a disadvantaged, high-migrant district in France: A perinatal audit. | Wrong study design |
| Savona-Ventura | 2003 | The outcome of diabetic pregnancies in Malta. | Wrong patient population |
| Schneider | 2003 | Pregnancy complicated by diabetic ketoacidosis: Maternal and fetal outcomes | Wrong patient population |
| Seimon | 2022 | Maternal and neonatal outcomes of women with gestational diabetes and without specific medical conditions: an Australian population-based study comparing induction of labor with expectant management | Wrong intervention |
| Sepe | 1985 | Gestational diabetes. Incidence, maternal characteristics, and perinatal outcome. | Wrong outcomes |
| Sergienko | 2011 | Risk factors for intrauterine fetal death (1988-2009) | Wrong outcomes |
| Setji | 2005 | Gestational diabetes mellitus | Wrong study design |
| Shaaban | 2006 | Associated risk factors with ante-partum intra-uterine fetal death | Wrong outcomes |
| Shahriari | 2023 | Maternal and fetal outcomes of pregnancies associated with single versus double abnormal values in 100 gr glucose tolerance test | Wrong intervention |
| Sharma | 2023 | Burden, Differentials and Causes of Stillbirths in India: A Systematic Review and Meta Analysis | Wrong study design |
| Shen | 2018 | Association Between Maternal Hyperglycemia and Composite Maternal-Birth Outcomes. | Data unavailable |
| Shen | 2025 | Universal screening for hyperglycemia in early pregnancy and the risk of adverse pregnancy outcomes | Wrong outcomes |
| Shoham | 2001 | Gestational diabetes complicated by hydramnios was not associated with increased risk of perinatal morbidity and mortality. | Wrong comparator |
| Silva | 2017 | Frequency of intra-uterine fetal deaths in Zonal hospital of Puerto Madryn (Argentina) during the period 2014-2016 | No full text available |
| Simmons | 2024 | Perinatal Outcomes in Early and Late Gestational Diabetes Mellitus After Treatment From 24–28 Weeks’ Gestation: A TOBOGM Secondary Analysis | Wrong outcomes |
| Simsek | 2021 | Do patients with a single abnormal OGTT value need a globally admitted definition such as "borderline GDM"? Pregnancy outcomes of these women and the evaluation of new inflammatory markers. | Wrong study design |
| Smulian | 2002 | Fetal deaths in the United States: Influence of high-risk conditions and implications for management | Wrong comparator |
| Sobande | 2005 | Complications of pregnancy and foetal outcomes in pregnant diabetic patients managed in a tertiary hospital in Saudi Arabia | Wrong outcomes |
| Sohn | 2020 | Delayed diagnosis of gestational diabetes mellitus and perinatal outcomes in women with large for gestational age fetuses during the third trimester | Wrong outcomes |
| Souza | 2018 | Maternal-fetal outcomes of pregnant women with and without gestational diabetes mellitus (GDM) | No full text available |
| Souza Stork | 2024 | Gestational Outcomes Related to the Occurrence of Gestational Diabetes Mellitus: A Cohort Study | Wrong outcomes |
| Sperling | 2018 | Prenatal Care Adherence and Neonatal Intensive Care Unit Admission or Stillbirth among Women with Gestational and Preexisting Diabetes Mellitus | Wrong setting |
| Srinivas | 2019 | Pregnancy outcomes in women with gestational diabetes at Leeds teaching hospital trust | Wrong study design |
| Srivastava | 2015 | Evaluation of adverse effects of hyperglycaemia on pregnancies in the maternity unit of Mafraq Hospital | Wrong comparator |
| Stacey | 2017 | Raised fasting plasma glucose and diagnosis of gestational diabetes in relation to risk of late stillbirth | Wrong setting |
| Stacey | 2019 | Gestational diabetes and the risk of late stillbirth: a case-control study from England, UK. | Wrong study design |
| Starikov | 2015 | Stillbirth in the pregnancy complicated by diabetes. | Wrong patient population |
| Stella | 2008 | The Coexistence of Gestational Hypertension and Diabetes: Influence on Pregnancy Outcome | Wrong comparator |
| Stogianni | 2019 | Obstetric and perinatal outcomes in pregnancies complicated by diabetes, and control pregnancies, in Kronoberg, Sweden. | Wrong outcomes |
| Subramanian | 2018 | Fetal outcome in pregnancies complicated with gestational diabetes mellitus | Wrong study design |
| Subramanian | 2010 | Pregnancy outcomes among minority women with gestational diabetes | Wrong comparator |
| Sudharshana Murthy | 2018 | Evaluation of Oxidative Stress and Proinflammatory Cytokines in Gestational Diabetes Mellitus and Their Correlation with Pregnancy Outcome. | Wrong comparator |
| Suwannarat | 2023 | Factors Associated with Cesarean Operations of Gestational Diabetic Mellitus and Diabetes Complications | Wrong study design |
| Su | 2021 | Increasing trend in the prevalence of gestational diabetes mellitus in Taiwan | Wrong outcomes |
| Swaminathan | 2010 | Metformin in gestational diabetes: An observational study | Wrong study design |
| Syed | 2011 | Effect of screening and management of diabetes during pregnancy on stillbirths. | Wrong study design |
| Szmuilowicz | 2019 | Gestational Diabetes Mellitus | Wrong study design |
| Tabatabaee | 2020 | Risk of Stillbirth in Women with Gestational Diabetes and High Blood Pressure. | Wrong study design |
| Tan | 2023 | Association between pregnancy-related factors and stillbirth: a retrospective cohort study based on 500 000 pregnant residents in Wuhan | Wrong outcomes |
| Tang | 2025 | Dose-response association between OGTT and adverse perinatal outcomes after IVF treatment: A cohort study based on a twin population | Data unavailable |
| Tennant | 2022 | Fasting plasma glucose, diagnosis of gestational diabetes and the risk of large for gestational age: a regression discontinuity analysis of routine data | Wrong comparator |
| Ticconi | 2020 | Pregnancy-Related Complications in Women with Recurrent Pregnancy Loss: A Prospective Cohort Study. | Wrong outcomes |
| Tieu | 2014 | Screening and subsequent management for gestational diabetes for improving maternal and infant health | Wrong study design |
| Tieu | 2017 | Dietary advice interventions in pregnancy for preventing gestational diabetes mellitus | Wrong study design |
| Tolefac | 2017 | Ten years analysis of stillbirth in a tertiary hospital in sub-Sahara Africa: A case control study | Wrong outcomes |
| Tsai | 2018 | Comparison of One-Step Versus Two Step Screening for Gestational Diabetes Mellitus and Adverse Obstetrical Outcomes at a Single Tertiary Center in Taiwan | Wrong comparator |
| Tundidor | 2010 | Perinatal outcome in women with gestational diabetes mellitus in relation with fetal sex | Wrong patient population |
| Turgal | 2013 | Intrauterine fetal demise: 9-years' experience at a tertiary hospital of turkey | Wrong study design |
| Uma | 2017 | Pregnancy outcome of gestational diabetes mellitus using a structured model of care : WINGS project (WINGS-10). | Wrong intervention |
| Vambergue | 1997 | Short, medium and long term consequences of gestational diabetes for mothers and infants | Wrong outcomes |
| Van-de-l’Isle | 2021 | Impact of changes to national UK Guidance on testing for gestational diabetes screening during a pandemic: a single-centre observational study | Wrong study design |
| Van Zyl | 2017 | Pregnancy outcome in patients attending Groote Schuur Hospital with pregestational and gestational diabetes | Wrong patient population |
| Vanlalhruaii | 2018 | How safe is metformin when initiated in early pregnancy? A retrospective 5-year study of pregnant women with gestational diabetes mellitus from India. | Wrong intervention |
| Varghese | 2012 | The prevalence, risk factors, maternal and fetal outcomes in gestational diabetes mellitus | Wrong outcomes |
| Volkov | 2023 | Stillbirth and fetal growth restriction | Wrong study design |
| Wallenstein | 2018 | Prematurity and Stillbirth | Wrong study design |
| Wang | 2013 | Incidence of adverse outcomes associated with gestational diabetes mellitus in low- and middle-income countries | Wrong study design |
| Wang | 2014 | Impact of gestational diabetes mellitus on pregnant, obstetric and neonatal outcomes: A 10-year retrospective double cohort study | Wrong outcomes |
| Wang | 2023 | Clinical analysis of diabetes in pregnancy with stillbirth | Wrong study design |
| Weeks | 1994 | Gestational diabetes: does the presence of risk factors influence perinatal outcome?. | Wrong outcomes |
| Wendland | 2011 | Lesser than diabetes hyperglycemia in pregnancy is related to perinatal mortality: a cohort study in Brazil. | Wrong outcomes |
| Wiser | 2008 | Gestational diabetes insipidus and intrauterine fetal death of monochorionic twins. | Wrong study design |
| Wolfson | 2022 | Levels, Trends and Risk Factors for Stillbirths in the United States: 2000-2017 | Wrong outcomes |
| Wolka | 2022 | Effect of diabetes mellitus on pregnancy and birth outcomes in Wolaita Zone, Southern Ethiopia: A retrospective cohort study | Wrong outcomes |
| Wood | 2003 | The risk of stillbirth in pregnancies before and after the onset of diabetes. | Wrong patient population |
| Wood | 2020 | Stillbirth and large for gestational age at birth. | Wrong outcomes |
| Wu | 2022 | Effect of Maternal Glucose and Triglyceride Levels during Early Pregnancy on Pregnancy Outcomes: A Retrospective Cohort Study. | Wrong outcomes |
| Yao | 2017 | The interaction between obesity and gestational diabetes on the risk of stillbirth | Data unavailable |
| Yao | 2018 | 758: Obesity and stillbirth revisited: the effects from gestational hypertension and gestational diabetes. | Wrong outcomes |
| Ye | 2022 | Gestational diabetes mellitus and adverse pregnancy outcomes: systematic review and meta-analysis | Wrong study design |
| Yogev | 2011 | Metabolic syndrome and gestational diabetes: The impact of achieving desired level of glycemic control on adverse pregnancy outcome | Wrong patient population |
| Yu | 2009 | Risk factors analysis of intrauterine fetal death in the third trimester | Wrong patient population |
| Zhang | 2021 | Factors Associated with Gestational Diabetes Mellitus: A Meta-Analysis. | Wrong study design |
| Zhao | 2021 | Effects of gestational diabetes mellitus and mid-pregnancy blood glucose levels on gestational weeks based on birth cohort study | Wrong outcomes |
| Zhu | 2015 | Pregnancy outcomes of gestational diabetes mellitus and hypothyroidism during pregnancy in a large Chinese cohort | Wrong patient population |
| Zilw | 2019 | Maternal Risk Factors for Stillbirth: A Registry–Based Study | Wrong study design |
| Zizzo | 2022 | Home management by remote self-monitoring in intermediate- and high-risk pregnancies: A retrospective study of 400 consecutive women. | Wrong study design |

**Unadjusted results and funnel plots**

Our main analysis was using adjusted results, however, to incorporate the whole body of literature, and to establish if our findings from the main adjusted analysis were also supported in the unadjusted data, we have included a meta-analysis of the results from the larger number of studies which presented unadjusted data.

# Supplementary figure 1: Random effects meta-analysis of unadjusted results from cohort studies by country income level

**
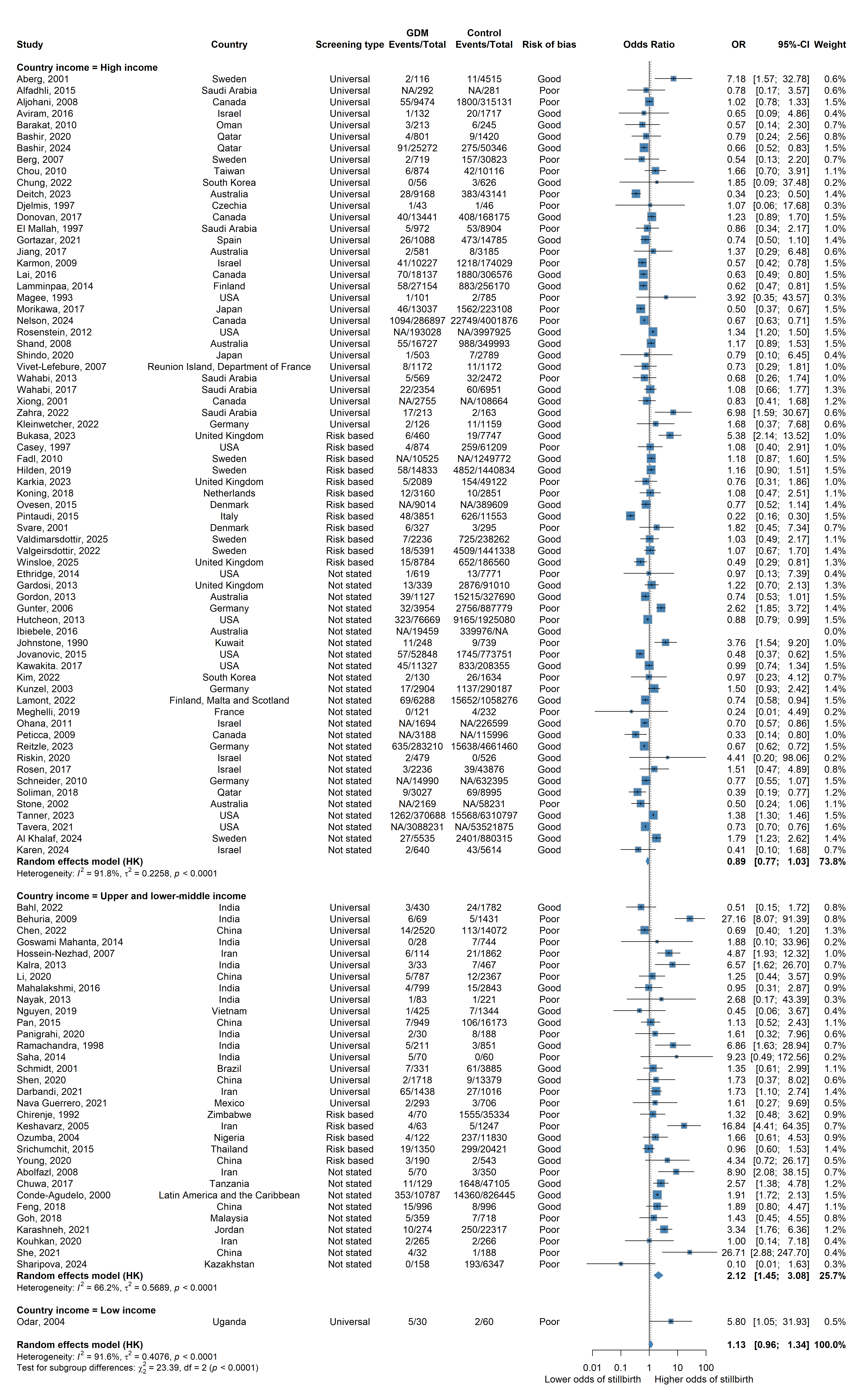
**

# Supplementary figure 2: Random effects meta-analysis of unadjusted results from cohort studies without continuity correction applied (sensitivity analysis) and therefore excluding studies with zero events in one or both arms

**
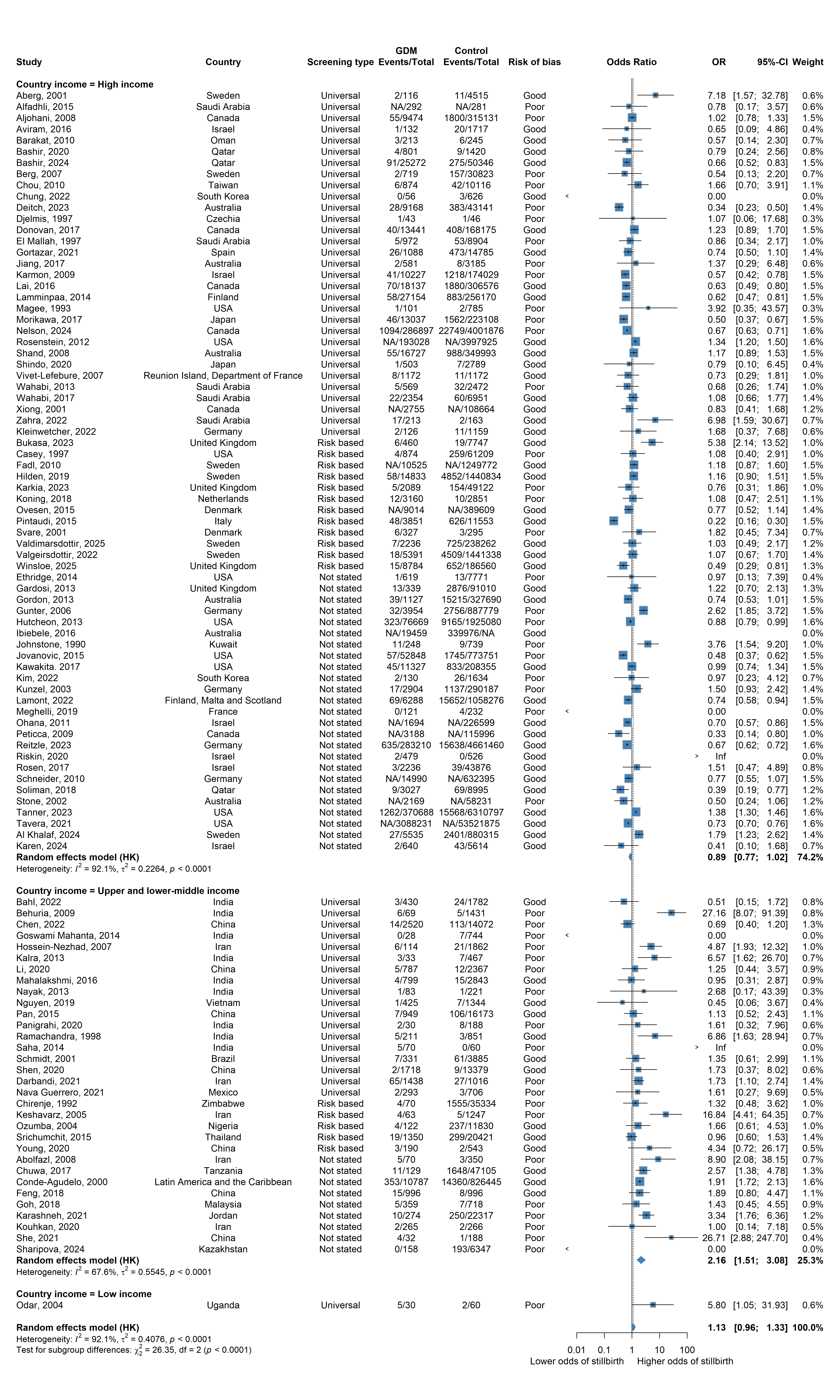
**

## Supplementary figure 3: Funnel plot of adjusted results from cohort studies


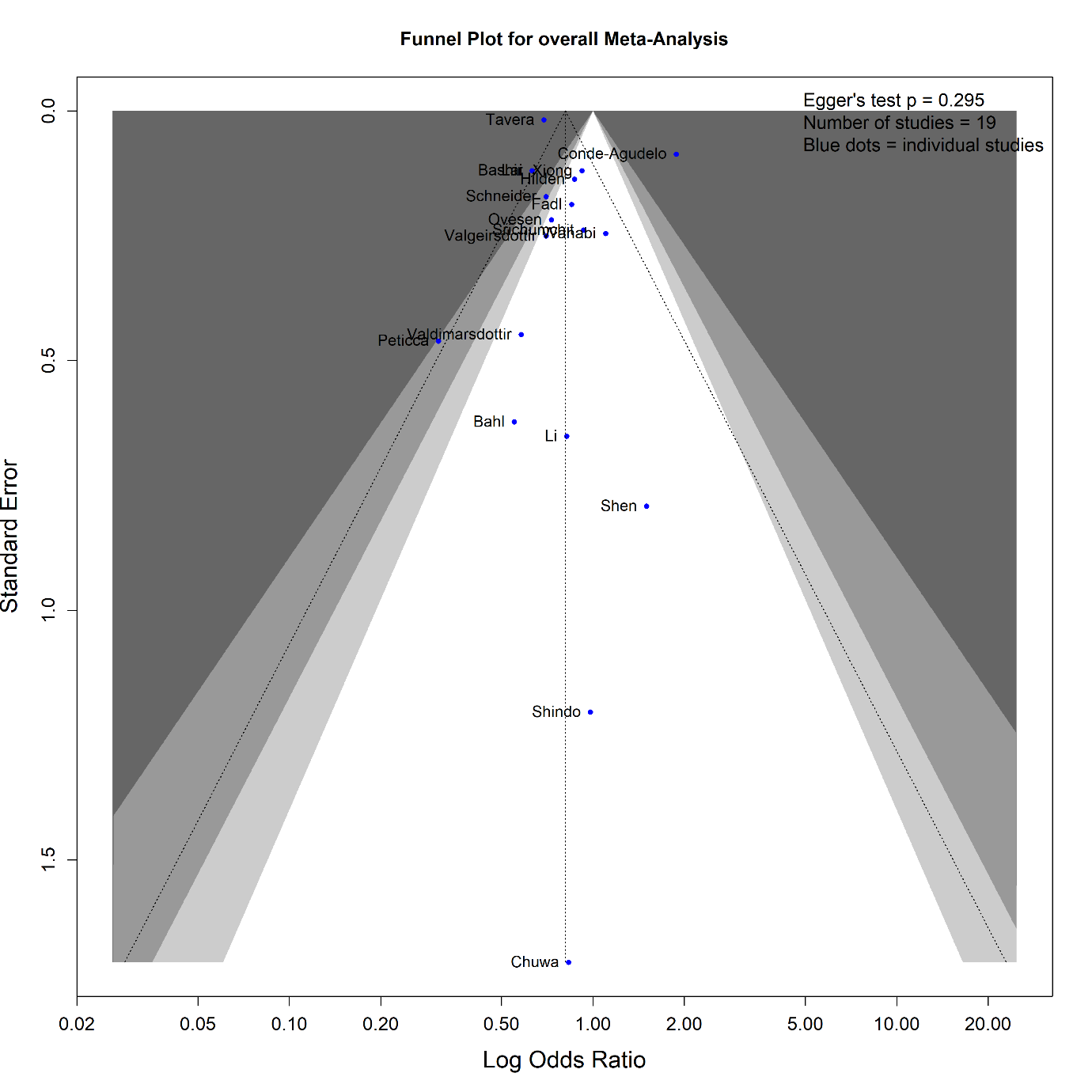


## Supplementary figure 4: Funnel plot of adjusted results from cohort studies restricted to high income countries


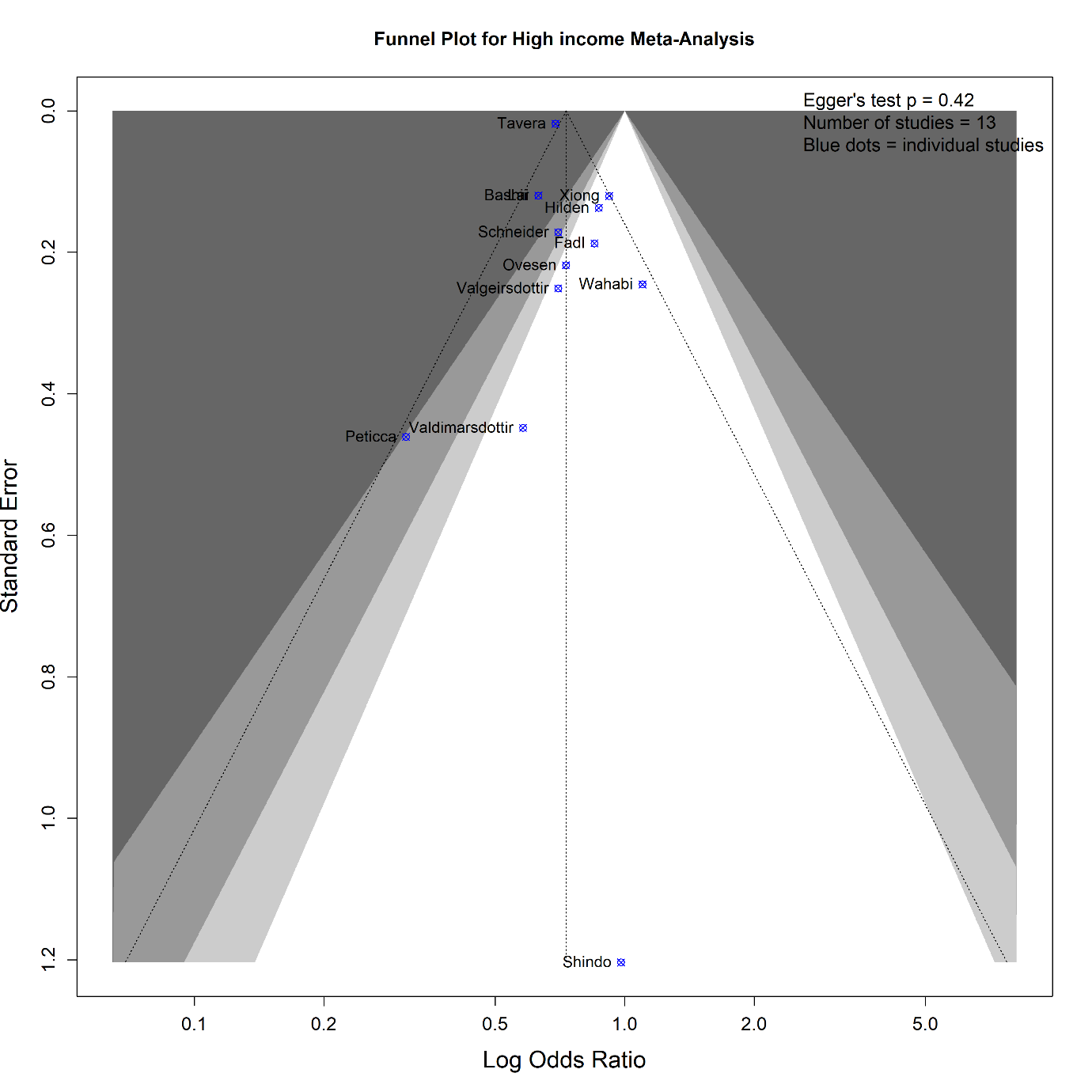


## Supplementary figure 5: Funnel plot of adjusted results from cohort studies restricted to upper and lower middle income countries


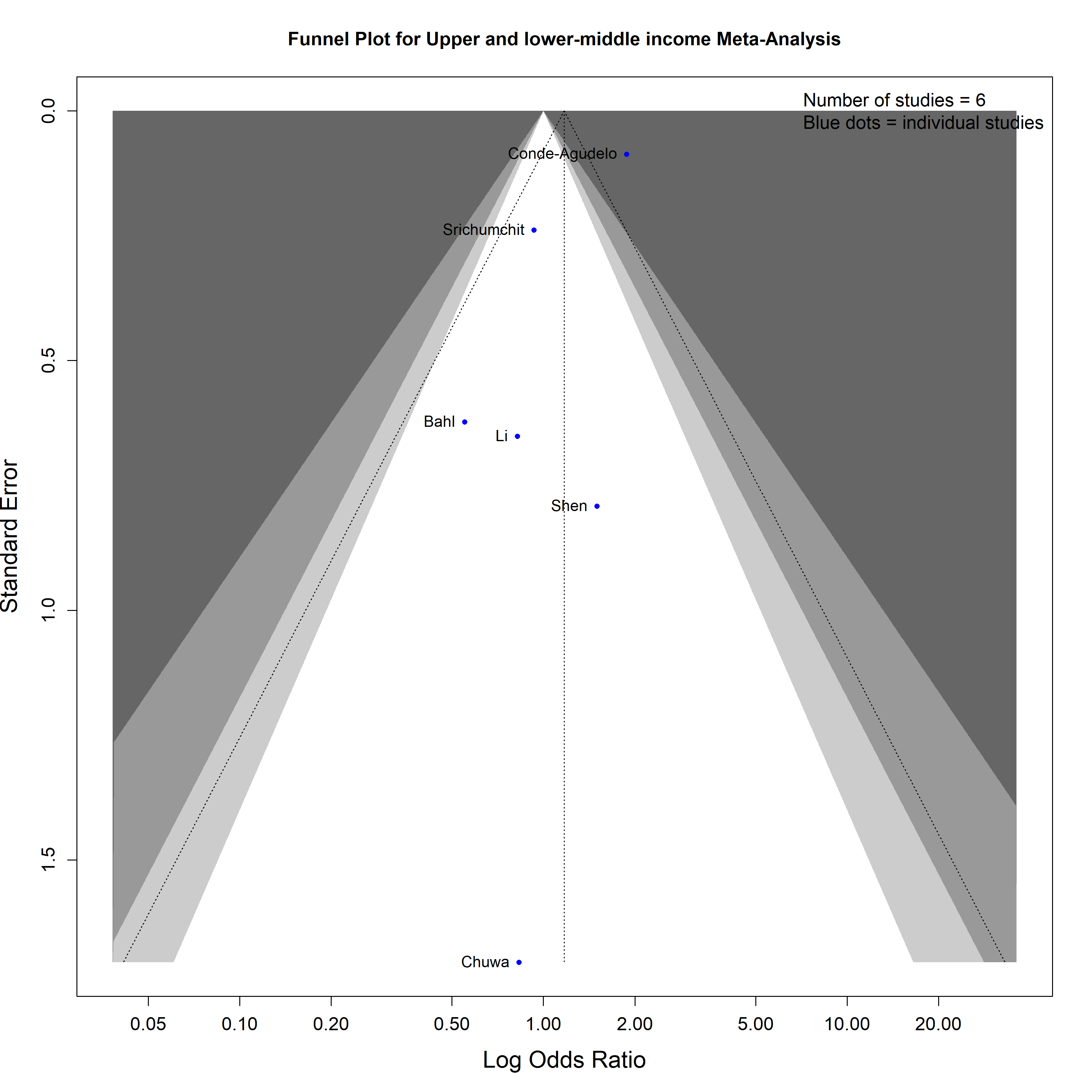


## Search strategy

( ALL ( ( gestational AND diabetes OR "pregnancy diabetes mellitus" ) AND ( "f*etal death" OR stillbirth ) ) OR TITLE-ABS-KEY ( ( "gestational diabetes" OR "pregnancy induced diabetes" OR "gestational diabetes mellitus" OR "maternal hypergly*emia" ) AND ( "intra-uterine death" OR "intra-uterine demise" OR "Intrauterine death" OR "intrauterine demise" OR stillbirth* OR stillborn* OR "f*etal loss" OR "f*etal demise" OR "f*etal death" OR "f*etus loss" OR "f*etus death" OR "f*etus demise" OR iud OR iufd ) AND NOT ( coil OR device OR mirena ) ) )

| **Search terms** |
| --- |
| Gestational diabetes |
| Pregnancy induced diabetes |
| Gestational diabetes mellitus |
| Maternal hyperglyc?emia |
| Pregnancy diabetes mellitus |
| F?etal death |
| Intra-uterine adju. (death or demise) |
| Intrauterine adju. (death or demise) |
| Stillbirth* |
| Stillborn* |
| F?etal or f?etus (loss or death or demise) |
| IUD |
| IUFD |
| NOT coil or device or mirena |

## Reference list of included studies.

1. Åberg A, Rydhstroem H, Frid A. Impaired glucose tolerance associated with adverse pregnancy outcome: A population-based study in southern Sweden. *AJOG*. 2001;184(2):77-83.

2. Abolfazl M, Hamidreza TS, Narges M, Maryam Y. Gestational diabetes and its association with unpleasant outcomes of pregnancy. Pakistan Journal of Medical Sciences. 2008;24:566-70.

3. Al Khalaf SY, Heazell Alexander EP, Kublickas Marius, Kublickiene Karolina, Khashan Ali S. Risk of stillbirth after a previous caesarean delivery: A Swedish nationwide cohort study. BJOG: An International Journal of Obstetrics & Gynaecology. 2024;131(8):1054-61.

4. Alfadhli EM, Osman EN, Basri TH, Mansuri NS, Youssef MH, Assaaedi SA, et al. Gestational diabetes among Saudi women: prevalence, risk factors and pregnancy outcomes. Ann Saudi Med. 2015;35(3):222-30.

5. Aljohani N, Rempel BM, Ludwig S, Morris M, Cheang M, Murray R, et al. Impact of diabetes on maternal-fetal outcomes in Manitoba: Relationship with ethnic and environmental factors. Clin Invest Med. 2008;31(6):E338-45.

6. Aviram A, Guy L, Ashwal E, Hiersch L, Yogev Y, Hadar E. Pregnancy outcome in pregnancies complicated with gestational diabetes mellitus and late preterm birth. Diabetes Res Clin Pract. 2016;113:198-203.

7. Bahl S, Dhabhai N, Taneja S, Mittal P, Dewan R, Kaur J, et al. Burden, risk factors and outcomes associated with gestational diabetes in a population-based cohort of pregnant women from North India. BMC Pregnancy and Childbirth. 2022;22(1).

8. Barakat MN, Youssef RM, Al-Lawati JA. Pregnancy outcomes of diabetic women: charting Oman's progress towards the goals of the Saint Vincent Declaration. Ann Saudi Med. 2010;30(4):265-70.

9. Bashir M, Aboulfotouh M, Dabbous Z, Mokhtar M, Siddique M, Wahba R, et al. Metformin-treated-GDM has lower risk of macrosomia compared to diet-treated GDM- a retrospective cohort study. J Matern Fetal Neonatal Med. 2020;33(14):2366-71.

10. Bashir M, Ibrahim I, Beer S, Shahbic H, Eltaher F, Al-Mutawaa K, et al. Integrated care of diabetes during pregnancy: a Qatari nationwide cohort. eClinicalMedicine. 2024;72:102605.

11. Behuria S. Maternal and foetal outcome in gestational diabetes mellitus - a prospective study. *International Journal of Gynecology and Obstetrics*. 2009;107.

12. Berg M, Adlerberth A, Sultan B, Wennergren M, Wallin G. Early random capillary glucose level screening and multidisciplinary antenatal teamwork to improve outcome in gestational diabetes mellitus. Acta Obstetricia et Gynecologica Scandinavica. 2007;86(3):283-90.

13. Bukasa LL, Cortina‐Borja M, Peters H, Taylor GP, Thorne C. Gestational diabetes in women living with HIV in the UK and Ireland: insights from population‐based surveillance data. Journal of the International AIDS Society. 2023;26(4).

14. Casey BM, Lucas MJ, McIntire DD, Leveno KJ. Pregnancy outcomes in women with gestational diabetes compared with the general obstetric population. Obstet Gynecol. 1997;90(6):869-73.

15. Chen J, Wang Z, Wu W, Chen H, Zhong C, Liang L, et al. Clinical analysis of 2860 cases of diabetes in pregnancy: a single-center retrospective study. BMC Pregnancy and Childbirth. 2022;22(1).

16. Chirenje MZ. The effects of established and gestational diabetes on pregnancy outcome at Harare Maternity Hospital. Cent Afr J Med. 1992;38(5):179-81.

17. Chou CY, Lin CL, Yang CK, Yang WC, Lee FK, Tsai MS. Pregnancy outcomes of Taiwanese women with gestational diabetes mellitus: a comparison of Carpenter-Coustan and National Diabetes Data Group criteria. J Womens Health (Larchmt). 2010;19(5):935-9.

18. Chung YS, Moon H, Kim EH. Risk of obstetric and neonatal morbidity in gestational diabetes in a single institution: A retrospective, observational study. Medicine (Baltimore). 2022;101(39):e30777.

19. Chuwa FS, Mwanamsangu AH, Brown BG, Msuya SE, Senkoro EE, Mnali OP, et al. Maternal and fetal risk factors for stillbirth in Northern Tanzania: A registry-based retrospective cohort study. PLOS ONE. 2017;12(8):e0182250.

20. CONDE‐AGUDELO A, Belizán JM, DÍAZ‐ROSSELLO JL. Epidemiology of fetal death in Latin America. Acta obstetricia et gynecologica Scandinavica. 2000;79(5):371-8.

21. Darbandi M, Rezaeian S, Dianatinasab M, Yaghoobi H, Soltani M, Etemad K, et al. Prevalence of gestational diabetes and its association with stillbirth, preterm birth, macrosomia, abortion and cesarean delivery: a national prevalence study of 11 provinces in Iran. J Prev Med Hyg. 2021;62(4):E885-e91.

22. Deitch J, Yates CJ, Hamblin PS, Kevat D, Shahid I, Teale G, et al. Prevalence of gestational diabetes mellitus, maternal obesity and associated perinatal outcomes over 10 years in an Australian tertiary maternity provider. Diabetes Res Clin Pract. 2023;203:110793.

23. Djelmis J, Blajić J, Buković D, Pfeifer D, Ivanisević M, Kendić S, et al. Glycosylated hemoglobin and fetal growth in normal, gestational and insulin dependent diabetes mellitus pregnancies. Coll Antropol. 1997;21(2):621-9.

24. Donovan LE, Edwards AL, Savu A, Butalia S, Ryan EA, Johnson JA, et al. Population-Level Outcomes with a 2-Step Approach for Gestational Diabetes Screening and Diagnosis. Can J Diabetes. 2017;41(6):596-602.

25. El Mallah KO, Narchi H, Kulaylat NA, Shaban MS. Gestational and pre-gestational diabetes: comparison of maternal and fetal characteristics and outcome. Int J Gynaecol Obstet. 1997;58(2):203-9.

26. Ethridge JK, Catalano PM, Waters TP. Perinatal Outcomes Associated With the Diagnosis of Gestational Diabetes Made by The International Association of the Diabetes and Pregnancy Study Groups Criteria. Obstetrics &amp; Gynecology. 2014;124(3):571-8.

27. Fadl HE, Ostlund IK, Magnuson AF, Hanson US. Maternal and neonatal outcomes and time trends of gestational diabetes mellitus in Sweden from 1991 to 2003. Diabet Med. 2010;27(4):436-41.

28. Feng R, Liu L, Zhang YY, Yuan ZS, Gao L, Zuo CT. Unsatisfactory Glucose Management and Adverse Pregnancy Outcomes of Gestational Diabetes Mellitus in the Real World of Clinical Practice: A Retrospective Study. Chin Med J (Engl). 2018;131(9):1079-85.

29. Gardosi J, Madurasinghe V, Williams M, Malik A, Francis A. Maternal and fetal risk factors for stillbirth: population based study. BMJ. 2013;346(jan24 3):f108-f.

30. Goh S, Rosilawati W, Hamid N. The influence of maternal gestational diabetes mellitus status and foetal sex on infant outcomes: a registry audit. International Journal of Public Health and Clinical Sciences. 2018;5(1):83-96.

31. Gordon A, Raynes-Greenow C, McGeechan K, Morris J, Jeffery H. Risk factors for antepartum stillbirth and the influence of maternal age in New South Wales Australia: A population based study. BMC Pregnancy and Childbirth. 2013;13(1):12.

32. Gortazar L, Flores-Le Roux JA, Benaiges D, Sarsanedas E, Navarro H, Payà A, et al. Trends in Prevalence of Diabetes among Twin Pregnancies and Perinatal Outcomes in Catalonia between 2006 and 2015: The DIAGESTCAT Study. Journal of Clinical Medicine. 2021;10(9):1937.

33. Goswami Mahanta T, Deuri A, Mahanta BN, Bordoloi P, Rasaily R, Mahanta J, et al. Maternal and foetal outcome of gestational diabetes mellitus in a rural block of Assam, India. Clinical Epidemiology and Global Health. 2014;2(1):9-15.

34. Günter HH, Tzialidou I, Scharf A, Wenzlaff P, Maul H, Hillemanns P. Intrauterine fetal death in pregnancies of women with preconceptional and gestational diabetes mellitus and of women without glucose tolerance disorders. Results of the perinatal registry of Lower Saxony, Germany. Z Geburtshilfe Neonatol. 2006.

35. Hildén K, Hanson U, Persson M, Magnuson A, Simmons D, Fadl H. Gestational diabetes and adiposity are independent risk factors for perinatal outcomes: a population based cohort study in Sweden. Diabetic Medicine. 2019;36(2):151-7.

36. Hossein-Nezhad A, Maghbooli Z, Vassigh A-R, Larijani B. Prevalence of Gestational Diabetes Mellitus and Pregnancy Outcomes in Iranian Women. Taiwanese Journal of Obstetrics and Gynecology. 2007;46(3):236-41.

37. Hutcheon JA, Kuret V, Joseph KS, Sabr Y, Lim K. Immortal time bias in the study of stillbirth risk factors: the example of gestational diabetes. Epidemiology. 2013;24(6):787-90.

38. Ibiebele I, Coory M, Smith GCS, Boyle FM, Vlack S, Middleton P, et al. Gestational age specific stillbirth risk among Indigenous and non-Indigenous women in Queensland, Australia: a population based study. BMC Pregnancy and Childbirth. 2016;16(1).

39. Jiang S, Chipps D, Cheung WN, Mongelli M. Comparison of adverse pregnancy outcomes based on the new IADPSG 2010 gestational diabetes criteria and maternal body mass index. Aust N Z J Obstet Gynaecol. 2017;57(5):533-9.

40. Johnstone FD, Nasrat AA, Prescott RJ. The effect of established and gestational diabetes on pregnancy outcome. Br J Obstet Gynaecol. 1990;97(11):1009-15.

41. Jovanovič L, Liang Y, Weng W, Hamilton M, Chen L, Wintfeld N. Trends in the incidence of diabetes, its clinical sequelae, and associated costs in pregnancy. Diabetes/Metabolism Research and Reviews. 2015;31(7):707-16.

42. Kalra P, Kachhwaha CP, Singh HV. Prevalence of gestational diabetes mellitus and its outcome in western Rajasthan. Indian J Endocrinol Metab. 2013;17(4):677-80.

43. Karasneh RA, Migdady FH, Alzoubi KH, Al-Azzam SI, Khader YS, Nusair MB. Trends in maternal characteristics, and maternal and neonatal outcomes of women with gestational diabetes: A study from Jordan. Ann Med Surg (Lond). 2021;67:102469.

44. Karen Baraah Abu, Steiner Naama, Rotem Reut, Baumfeld Yael, Weintraub AY, Eshkoli Tamar. Perinatal outcomes in pregnancies achieved after fertility treatments with and without diet-controlled GDM. Archives of Gynecology and Obstetrics. 2024;310(6):2899-906.

45. Karkia R, Giacchino T, Shah S, Gough A, Ramadan G, Akolekar R. Gestational Diabetes Mellitus: Association with Maternal and Neonatal Complications. Medicina. 2023;59(12):2096.

46. Karmon A, Levy A, Holcberg G, Wiznitzer A, Mazor M, Sheiner E. Decreased perinatal mortality among women with diet-controlled gestational diabetes mellitus. Int J Gynaecol Obstet. 2009;104(3):199-202.

47. Kawakita T, Bowers K, Hazrati S, Zhang C, Grewal J, Chen Z, et al. Increased Neonatal Respiratory Morbidity Associated with Gestational and Pregestational Diabetes: A Retrospective Study. American Journal of Perinatology. 2017;34(11):1160-8.

48. Keshavarz M, Cheung NW, Babaee GR, Moghadam HK, Ajami ME, Shariati M. Gestational diabetes in Iran: incidence, risk factors and pregnancy outcomes. Diabetes Res Clin Pract. 2005;69(3):279-86.

49. Kim Y, Hong SY, Kim SY, Kim YM, Sung JH, Choi SJ, et al. Obstetric and neonatal outcomes of gestational diabetes mellitus in twin pregnancies according to changes in its diagnostic criteria from National Diabetes Data Group criteria to Carpenter and Coustan criteria: a retrospective cohort study. BMC Pregnancy Childbirth. 2022;22(1):9.

50. Kleinwechter Helmut J, Weber Katharina S, Mingers Nina, Ramsauer Babett, Schaefer-Graf Ute M, Groten Tanja, et al. Gestational diabetes mellitus and COVID-19: results from the COVID-19&#x2013;Related&#xa0;Obstetric and Neonatal Outcome Study (CRONOS). American Journal of Obstetrics & Gynecology. 2022;227(4):631.e1-.e19.

51. Koning SH, Van Zanden JJ, Hoogenberg K, Lutgers HL, Klomp AW, Korteweg FJ, et al. New diagnostic criteria for gestational diabetes mellitus and their impact on the number of diagnoses and pregnancy outcomes. Diabetologia. 2018;61(4):800-9.

52. Kouhkan A, Najafi L, Malek M, Baradaran HR, Hosseini R, Khajavi A, et al. Gestational diabetes mellitus: Major risk factors and pregnancy-related outcomes: A cohort study. Int J Reprod Biomed. 2021;19(9):827-36.

53. Künzel W, Misselwitz B. Unexpected fetal death during pregnancy--a problem of unrecognized fetal disorders during antenatal care? Eur J Obstet Gynecol Reprod Biol. 2003;110 Suppl 1:S86-92.

54. Lai FY, Johnson JA, Dover D, Kaul P. Outcomes of singleton and twin pregnancies complicated by pre-existing diabetes and gestational diabetes: A population-based study in Alberta, Canada, 2005-11. J Diabetes. 2016;8(1):45-55.

55. Lamminpää R, Vehviläinen-Julkunen K, Gissler M, Selander T, Heinonen S. Pregnancy outcomes in women aged 35 years or older with gestational diabetes - a registry-based study in Finland. J Matern Fetal Neonatal Med. 2016;29(1):55-9.

56. Lamont K, Scott NW, Gissler M, Gatt M, Bhattacharya S. Risk of Recurrent Stillbirth in Subsequent Pregnancies. Obstet Gynecol. 2022;139(1):31-40.

57. Li MF, Ma L, Yu TP, Zhu Y, Chen MY, Liu Y, et al. Adverse maternal and neonatal outcomes in pregnant women with abnormal glucose metabolism. Diabetes Res Clin Pract. 2020;161:108085.

58. Magee MS, Walden CE, Benedetti TJ, Knopp RH. Influence of diagnostic criteria on the incidence of gestational diabetes and perinatal morbidity. International Journal of Gynecology & Obstetrics. 1993;42(3):327-8.

59. Mahalakshmi MM, Bhavadharini B, Maheswari K, Kalaiyarasi G, Anjana RM, Ranjit U, et al. Comparison of maternal and fetal outcomes among Asian Indian pregnant women with or without gestational diabetes mellitus: A situational analysis study (WINGS-3). Indian J Endocrinol Metab. 2016;20(4):491-6.

60. Meghelli L, Vambergue A, Drumez E, Deruelle P. Complications of pregnancy in morbidly obese patients: What is the impact of gestational diabetes mellitus? J Gynecol Obstet Hum Reprod. 2020;49(1):101628.

61. Morikawa M, Sugiyama T, Sagawa N, Hiramatsu Y, Ishikawa H, Hamada H, et al. Perinatal mortality in Japanese women diagnosed with gestational diabetes mellitus and diabetes mellitus. J Obstet Gynaecol Res. 2017;43(11):1700-7.

62. Nava Guerrero EN, Salcedo González A, Hernández Escobar CE, Rivera FC, Mondragón Ortiz EJ, De Alba IM, et al. Prevalence, risk factors and perinatal outcomes of gestational diabetes in Mexican adolescents when applying diagnostic criteria from three different international guidelines. International Journal of Diabetes in Developing Countries. 2021;41(2):212-20.

63. Nayak PK, Mitra S, Sahoo JP, Daniel M, Mathew A, Padma A. Feto-maternal outcomes in women with and without gestational diabetes mellitus according to the International Association of Diabetes and Pregnancy Study Groups (IADPSG) diagnostic criteria. Diabetes Metab Syndr. 2013;7(4):206-9.

64. Nelson CR, Dzakpasu S, Moore AM, Darling EK, Edwards W, Murphy P, et al. Diabetes mellitus in pregnancy across Canada. BMC Pregnancy and Childbirth. 2024;24(1).

65. Nguyen CL, Lee AH, Minh Pham N, Hoang Nguyen PT, Ha AVV, Khac Chu T, et al. Prevalence and pregnancy outcomes of gestational diabetes mellitus by different international diagnostic criteria: a prospective cohort study in Vietnam. J Matern Fetal Neonatal Med. 2020;33(21):3706-12.

66. Odar E, Wandabwa J, Kiondo P. Maternal and fetal outcome of gestational diabetes mellitus in Mulago Hospital, Uganda. Afr Health Sci. 2004;4(1):9-14.

67. Ohana O, Holcberg G, Sergienko R, Sheiner E. Risk factors for intrauterine fetal death (1988-2009). J Matern Fetal Neonatal Med. 2011;24(9):1079-83.

68. Ovesen PG, Jensen DM, Damm P, Rasmussen S, Kesmodel US. Maternal and neonatal outcomes in pregnancies complicated by gestational diabetes. a nation-wide study. J Matern Fetal Neonatal Med. 2015;28(14):1720-4.

69. Ozumba BC, Obi SN, Oli JM. Diabetes mellitus in pregnancy in an African population. Int J Gynaecol Obstet. 2004;84(2):114-9.

70. Pan L, Leng J, Liu G, Zhang C, Liu H, Li M, et al. Pregnancy outcomes of Chinese women with gestational diabetes mellitus defined by the IADPSG's but not by the 1999 WHO's criteria. Clin Endocrinol (Oxf). 2015;83(5):684-93.

71. Panigrahi A, Mallicka, Panda J. Gestational diabetes mellitus, its associated factors, and the pregnancy outcomes among pregnant women attending tertiary care hospitals of Bhubaneswar, India. International Journal of Diabetes in Developing Countries. 2020;40(3):371-8.

72. Peticca P, Keely EJ, Walker MC, Yang Q, Bottomley J. Pregnancy outcomes in diabetes subtypes: how do they compare? A province-based study of Ontario, 2005-2006. J Obstet Gynaecol Can. 2009;31(6):487-96.

73. Pintaudi B, Lucisano G, Pellegrini F, D’Ettorre A, Lepore V, De Berardis G, et al. The long-term effects of stillbirth on women with and without gestational diabetes: a population-based cohort study. Diabetologia. 2015;58(1):67-74.

74. Ramachandran A, Snehalatha C, Clementina M, Sasikala R, Vijay V. Foetal outcome in gestational diabetes in south Indians. Diabetes Res Clin Pract. 1998;41(3):185-9.

75. Reitzle L, Heidemann C, Baumert J, Kaltheuner M, Adamczewski H, Icks A, et al. Pregnancy Complications in Women With Pregestational and Gestational Diabetes Mellitus. Dtsch Arztebl Int. 2023;120(6):81-6.

76. Riskin A, Itzchaki O, Bader D, Iofe A, Toropine A, Riskin-Mashiah S. Perinatal Outcomes in Infants of Mothers with Diabetes in Pregnancy. Isr Med Assoc J. 2020;22(9):569-75.

77. Rosen H, Shmueli A, Ashwal E, Hiersch L, Yogev Y, Aviram A. Delivery outcomes of large-for-gestational-age newborns stratified by the presence or absence of gestational diabetes mellitus. Int J Gynaecol Obstet. 2018;141(1):120-5.

78. Rosenstein MG, Cheng YW, Snowden JM, Nicholson JM, Doss AE, Caughey AB. The risk of stillbirth and infant death stratified by gestational age in women with gestational diabetes. American Journal of Obstetrics and Gynecology. 2012;206(4):309.e1-.e7.

79. Saha S, Biswas S, Mitra D, Adhikari A, Saha C. Histologic and morphometric study of human placenta in gestational diabetes mellitus. Ital J Anat Embryol. 2014;119(1):1-9.

80. Schmidt MI, Duncan BB, Reichelt AJ, Branchtein L, Matos MC, Costa E Forti A, et al. Gestational Diabetes Mellitus Diagnosed With a 2-h 75-g Oral Glucose Tolerance Test and Adverse Pregnancy Outcomes. Diabetes Care. 2001;24(7):1151-5.

81. Schneid‐Kofman N, Sheiner E, Levy A, Holcberg G. Risk factors for wound infection following cesarean deliveries. International Journal of Gynecology &amp; Obstetrics. 2005;90(1):10-5.

82. Shand AW, Bell JC, McElduff A, Morris J, Roberts CL. Outcomes of pregnancies in women with pre-gestational diabetes mellitus and gestational diabetes mellitus; a population-based study in New South Wales, Australia, 1998-2002. Diabet Med. 2008;25(6):708-15.

83. Sharipova M, Tanysheva G, Sharipova K, Ion B, Shakhanova A. Association between maternal physiological and pathological factors and the risk of stillbirth and perinatal mortality. World Academy of Sciences Journal. 2024;7(1).

84. She W, Li T, Liu Y, Liu X. CircRNA circVEGFC is Highly Expressed in Gestational Diabetes Mellitus (GDM) and It is Correlated with Multiple Adverse Events. Diabetes, Metabolic Syndrome and Obesity: Targets and Therapy. 2021;Volume 14:4409-14.

85. Shen Y, Jia Y, Zhou J, Cheng X-Y, Huang H-Y, Sun C-Q, et al. Association of gestational diabetes mellitus with adverse pregnancy outcomes: our experience and meta-analysis. International Journal of Diabetes in Developing Countries. 2020;40(3):357-70.

86. Shindo R, Aoki S, Kasai J, Saigusa Y, Nakanishi S, Miyagi E. Impact of introducing the International Association of Diabetes and Pregnancy Study Groups (IADPSG) criteria on pregnancy outcomes in Japan. Endocrine Journal. 2020;67(1):15-20.

87. Soliman A, Salama H, Al Rifai H, De Sanctis V, Al-Obaidly S, Al Qubasi M, et al. The effect of different forms of dysglycemia during pregnancy on maternal and fetal outcomes in treated women and comparison with large cohort studies. Acta Biomed. 2018;89(S5):11-21.

88. Srichumchit S, Luewan S, Tongsong T. Outcomes of pregnancy with gestational diabetes mellitus. Int J Gynaecol Obstet. 2015;131(3):251-4.

89. Stone CA, McLachlan KA, Halliday JL, Wein P, Tippett C. Gestational diabetes in Victoria in 1996: incidence, risk factors and outcomes. Medical Journal of Australia. 2002;177(9):486-91.

90. Svare JA, Hansen BB, Mølsted‐Pedersen L. Perinatal complications in women with gestational diabetes mellitus. Acta Obstetricia et Gynecologica Scandinavica. 2001;80(10):899-904.

91. Tanner D, Murthy S, Lavista Ferres JM, Ramirez J-M, Mitchell EA. Risk factors for late (28+ weeks’ gestation) stillbirth in the United States, 2014–2015. PLOS ONE. 2023;18(8):e0289405.

92. Tavera G, Dongarwar D, Salemi JL, Akindela O, Osazuwa I, Akpan EB, et al. Diabetes in pregnancy and risk of near-miss, maternal mortality and foetal outcomes in the USA: a retrospective cross-sectional analysis. J Public Health (Oxf). 2022;44(3):549-57.

93. Valdimarsdottir R, Vanky E, Elenis E, Ahlsson F, Lindström L, Junus K, et al. Polycystic ovary syndrome and gestational diabetes mellitus association to pregnancy outcomes: A national register‐based cohort study. Acta Obstetricia et Gynecologica Scandinavica. 2025;104(1):119-29.

94. Valgeirsdóttir I, Hanson U, Schwarcz E, Simmons D, Backman H. Diet-Treated Gestational Diabetes Mellitus Is an Underestimated Risk Factor for Adverse Pregnancy Outcomes: A Swedish Population-Based Cohort Study. Nutrients. 2022;14(16):3364.

95. Vivet-Lefébure A, Roman H, Robillard PY, Laffitte A, Hulsey TC, Camp G, et al. [Obstetrical and neonatal outcomes of gestational diabetes mellitus at Reunion Island (France)]. Gynecol Obstet Fertil. 2007;35(6):530-5.

96. Wahabi HA, Esmaeil SA, Fayed A, Alzeidan RA. Gestational diabetes mellitus: maternal and perinatal outcomes in King Khalid University Hospital, Saudi Arabia. J Egypt Public Health Assoc. 2013;88(2):104-8.

97. Wahabi H, Fayed A, Esmaeil S, Mamdouh H, Kotb R. Prevalence and Complications of Pregestational and Gestational Diabetes in Saudi Women: Analysis from Riyadh Mother and Baby Cohort Study (RAHMA). BioMed Research International. 2017;2017(1):6878263.

98. Winsloe C, Elhindi J, Vieira MC, Relph S, Arcus CG, Alagna A, et al. Differences in Factors Associated With Preterm and Term Stillbirth: A Secondary Cohort Analysis of the DESiGN Trial. BJOG: An International Journal of Obstetrics & Gynaecology. 2025;132(1):89-98.

99. Xiong X, Saunders LD, Wang FL, Demianczuk NN. Gestational diabetes mellitus: prevalence, risk factors, maternal and infant outcomes. Int J Gynaecol Obstet. 2001;75(3):221-8.

100. Young S-C, Yiu M-S, So P-L. Effect of new diagnostic criteria on detection and pregnancy outcomes of gestational diabetes mellitus: a retrospective study. Hong Kong Journal of Gynaecology, Obstetrics and Midwifery. 2020;20(1):16-21.

101. Zahra A, Hassan SU, Parveen N, Iqbal N, Batool A. Pregnancy related complications due to obesity and gestational diabetes mellitus among women in Saudi Arabia. Afr J Reprod Health. 2022;26(2):38-46.
